# Extent of Integration of Priority Interventions into General Health Systems: A Case Study of Neglected Tropical Diseases Programme in the Western Region of Ghana

**DOI:** 10.1371/journal.pntd.0004725

**Published:** 2016-05-20

**Authors:** Ernest O. Mensah, Moses K. Aikins, Margaret Gyapong, Francis Anto, Moses J. Bockarie, John O. Gyapong

**Affiliations:** 1 School of Public Health, College of Health Sciences, University of Ghana, Accra, Ghana; 2 Dodowa Research Centre, Ghana Health Service, Dodowa, Ghana; 3 Vector Biology Department, Liverpool School of Tropical Medicine, Liverpool, United Kingdom; Case Western Reserve University School of Medicine, UNITED STATES

## Abstract

**Background:**

The global health system has a large arsenal of interventions, medical products and technologies to address current global health challenges. However, identifying the most effective and efficient strategies to deliver these resources to where they are most needed has been a challenge. Targeted and integrated interventions have been the main delivery strategies. However, the health system discourse increasingly favours integrated strategies in the context of functionally merging targeted interventions with multifunctional health care delivery systems with a focus on strengthening country health systems to deliver needed interventions. Neglected Tropical Diseases (NTD) have been identified to promote and perpetuate poverty hence there has been global effort to combat these diseases. The Neglected Tropical Diseases Programme (NTDP) in Ghana has a national programme team and office, however, it depends on the multifunctional health delivery system at the regional and district level to implement interventions. The NTDP seeks further health system integration to accelerate achievement of coverage targets. The study estimated the extent of integration of the NTDP at the national, regional and district levels to provide evidence to guide further integration.

**Methodology/Principal Findings:**

The research design was a descriptive case study that interviewed key persons involved in the programme at the three levels of the health system as well as extensive document review. Integration was assessed on two planes—across health system functions–stewardship and governance, financing, planning, service delivery, monitoring and evaluation and demand generation; and across three administrative levels of the health system–national, regional and district. A composite measure of integration designated Cumulative Integration Index (CII) with a range of 0.00–1.00 was used to estimate extent of integration at the three levels of the health system. Service delivery was most integrated while financing and planning were least integrated. Extent of integration was partial at all levels of the health system with a CII of 0.48–0.68; however it was higher at the district compared to the national and regional levels.

**Conclusions/Significance:**

To ensure further integration of the NTDP, planning and finance management activities must be decentralized to involve regional and district levels of the health system. The study provides an empirical measure of extent of integration and indicators to guide further integration.

## Author Summary

Two main strategies have been used to address diseases that affects large sections of populations. One strategy called targeted or vertical programme sets up separate system from the general health system with its own human resources, management, implementation, data reporting and evaluation systems. Integrated (also called horizontal) strategy on the other hand uses existing health system structures to implement activities to control target health problems. Integrated strategy is preferred because it strengthens country health systems. The Neglected Tropical Diseases Programme (NTDP) in Ghana has a dedicated management structure at the national level but uses general health system structures at the regional and district levels to implement activities. This study assessed the extent of integration of the NTDP into the health system at the national, regional and district levels. It was found that the NTDP activities were better integrated at the district compared to the regional and national levels of the health system. Furthermore, it also found that service delivery activities were most integrated while financing and planning activities were least integrated at all levels of the health system. These findings provide points to guide efforts to make the NTDP more integrated and can be applied to other health programmes.

## Introduction

The debate on targeted and integrated strategies to health care delivery is long standing and continues to attract policy discourse though the dichotomy is considered simplistic and unrepresentative of reality for the reason that extremes of purely targeted or fully integrated interventions are exceptions; rather there is a continuum of integration of priority interventions across various levels of the health system [1–4]. Other terminologies for targeted and integrated are: vertical and horizontal; disease control and general health care approaches; and selective and comprehensive health systems [3]. The global health system landscape is characterized by several targeted programmes especially in developing countries where health care is delivered by a public health system mainly with state support aimed at providing basic health care services to the population. The evidence is that these health systems have significant targeted programmes that emphasize specific interventions focused on priority diseases affecting sections of the population. Continuous co-existence of the two service delivery strategies suggests importance of both strategies to achieving the goals of health systems–health, financial risk protection and responsiveness [5–7].

The Alma Ata Conference [8] which proposed primary health care and two important documents produced by the World Health Organization [9, 10] on indicators of health system performance and health system strengthening seemed to have tilted the debate towards integrated health system. They advocated the need to identify real drivers of effective health system and making efforts to strengthen them to deliver needed interventions to the general population. World Health Assembly (WHA) on the other hand, through its resolutions has been pivotal in providing direction for setting up of Global Health Initiatives (GHI) to control diseases of public health importance. The resolutions have set specific disease control agenda based on prevailing scientific evidence and proposed intervention end points—prevention, control, elimination or eradication [11–15]. These resolutions have been the rallying points for research, resource mobilization and programme design. Globally, successes in such GHI has been recorded in the eradication of smallpox, a real possibility of poliomyelitis and guinea worm eradication as well as a significant abatement of the rapid spread of HIV infection and associated morbidity and mortality in the last decade of the 20^th^ Century [1]. Put together, the WHA resolutions with associated resource mobilization to achieve its objectives and successes of GHI have been great advocates for targeted programmes characterizing the global public health landscape especially in developing countries. Global partnerships supporting specific GHI such as the Global Programme to Eliminate Lymphatic Filariasis (GPELF), Global Alliance for Vaccines and Immunization (GAVI), Drugs for Neglected Diseases initiative (DNDi), and Global Fund for HIV/AIDS, Malaria and TB have reinforced targeted interventions. However, the ideal way forward is the strengthening of an integrated health system not only to effectively deliver the plethora of effective interventions and resources available–essential medicines, skilled human resource, community mobilization techniques, diagnostic and monitoring tools—to affected target populations; but also achieve high intervention coverage [10].

The Neglected Tropical Diseases Programme (NTDP) has a programme office and staff at the National level but uses decentralized regional and district level health structures (i.e. health system human resource, organizational and management arrangements, implementation processes, data reporting channels, and evaluation systems etc.) to implement its activities. The study evaluated the extent of integration of the NTDP activities into the public health system.

### NTDP and the health system in Ghana

The past decade has seen global concerted effort to combat Neglected Tropical Diseases (NTDs) resulting in multiple global health organizations, donors, pharmaceutical companies, governments and researchers directing resources towards control of NTDs [16, 17]. The five most prevalent NTDs in Ghana–lymphatic filariasis (LF), onchocerciasis, schistosomiasis, soil transmitted helminths and trachoma—have gained the most attention. Lymphatic filariasis and trachoma are targeted for elimination while the others have control targets [18]. To maximize impact of increasing partnerships and resources dedicated to NTD control, four programme organizational strategies have been suggested. First, is coordination of partner activities at national and international levels. Secondly, programmes with similar delivery strategies should be integrated for delivery on the same platform. Thirdly, NTD programme activities must be integrated into the public health system to improve coverage; and finally there must be collaboration between NTD programmes and HIV/AIDS, TB and Malaria control programmes which are relatively well resourced to strengthen the public health system for the benefit of all [1]. Integration of NTD programme activities into health system structures appears to be the option relatively within the control of country NTD programmes. While the other options require policy change by funding agencies or merger of separate programmes with possibility of loss of authority by some programme management, and willingness of non-NTD programme managers to collaborate with NTD programmes; integration of NTDP activities into the health system depends largely on NTD programmes. The Neglected Tropical Diseases Programme in Ghana seeks increased health system integration to accelerate achievement of coverage targets [19].

The NTDP is responsible for implementing interventions (mass drug administration and morbidity management) for control of five NTDs in Ghana: Lymphatic filariasis, schistosomiasis, soil transmitted helminths, onchocerciasis and trachoma which are endemic in several communities in Ghana [19–21].There is geographic overlap of these diseases. The total population at risk of lymphatic filariasis was estimated at 12 million in 2009 [19]. Preventive chemotherapy through mass drug administration (MDA) provides ivermectin and albendazole annually to populations at risk of lymphatic filariasis. Ivermectin is administered annually or semi-annually in onchocerciasis endemic communities while Praziquantel and Albendazole or Mebendazole are administered especially to school age children for control of schistosomiasis and soil transmitted helminths. Azithromycin is administered to control trachoma [22].

At the national level the NTDP undertakes strategic planning, advocacy, monitoring and evaluation. However national, regional and district health system roles are not mutually exclusive. The NTDP is integrated to some extent but the nature and extent of integration at the various levels of the health system is not known. The vision of the Ghana NTDP is to “improve on the capacity of the Ghana Health Service (GHS) to establish an integrated NTD control programme capable of delivering interventions to prevent, control, eliminate or eradicate the neglected tropical diseases by the year 2020” [19]. This study assessed the extent of integration of the NTDP into the public health system using health system functions to provide intervention points for further integration.

### Conceptual framework

Integration in the health sector has been variously defined and applied to achieve ends broadly conceptualized as reducing fragmentation and duplication of interventions; aggregation of usually segmented health care units or specialties and related social services to achieve a continuum of health care, ease of access, improved health outcomes, wide population coverage, user satisfaction and efficiency [23–25]. Three main forms of integration can be identified. The first, integrated health care, provides a ‘one-stop shop’ health care service encompassing health promotion, diagnostics, treatment and rehabilitation by bringing interdependent service providers under one organizational structure or management to improve accessibility, efficiency, quality of care and client satisfaction [26–28]. The second, is the delivery of multiple population-based health interventions on a single platform for efficiency and improved population coverage [29, 30]. The simultaneous administration of multiple drugs through MDA to a population affected by any combination of NTDs is an example. The third form, which is the focus of this study, is “… a process where disease control activities are functionally merged or tightly coordinated with multifunctional health care delivery” [31]. It involves the merger and delivery of specific disease control interventions with public health system structures and resources to improve access and coverage [2, 32].

The study evaluates the extent of integration of the NTDP using an analytical framework by Atun et al [23]. The framework assesses the extent of integration of priority health intervention activities with respect to six health system functions—stewardship and governance, financing, planning, service delivery, monitoring and evaluation and demand generation. This framework was chosen for three main reasons. First, it was developed purposely to provide a consistent approach to evaluate the interface of priority health intervention programmes and health systems through the lens of integration. This is relevant to the study because the 5 NTDs are managed as priority interventions delivered through the structures of the public health system especially at the sub-national levels. Secondly, the framework has been applied successfully to examine Global Fund sponsored priority health programmes (HIV/AIDS, Malaria and TB) in several countries with respect to extent and nature of integration [23, 33–35]. Thirdly, the health system functions employed by the framework have universal application having been built on widely accepted health system function models [3, 5, 6, 9, 36].

The framework, presented in Table 1, identifies six functions of the health system designated as Critical Health System Functions. These are stewardship and governance, financing, planning, service delivery, demand generation and monitoring and evaluation. The critical health system functions are similar to the WHO health system building blocks and functions described in other health system frameworks [5, 9, 10, 36, 37]. Each health system function is made up of elements which constitute defined activities classified under the function. A health system function is considered integrated when health system structures are applied to implement elements of the health system function. When distinct structures are used to implement elements of a health system function for the health system and the targeted intervention programme it is considered to have non-existent integration. On the other hand when elements of a health system function are implemented by both health system and the targeted intervention structures it is assessed as partial integration. The mean extent of integration of health system elements is assigned as the extent of integration for the health system function. A composite score of all six health system functions is used to estimate the extent of integration of the priority intervention at each health system level—national, regional and district.

**Table 1:** Health system functions, elements and extent of integration.

Critical Health System Functions	Elements of Health System Function	Extent of integration of health system functions		
		Full	Partial	Non-existent
**Stewardship and Governance**	1. Accountability	Governance arrangement for NTD same for public health system	Governance shared by public health system and NTD specific structure	Governance involves only NTD specific unit
	2. Reporting			
	3. Performance management			
**Financing**	1. Pooling of funds	Fully funded through general health service budget	Funding by earmarked fund from government or donor agency channelled through health system	Funds provided directly through NTD programme to address only NTD issues
**Planning**	1. Needs assessment	When elements are undertaken by public health system structures	When elements decision taken by NTD managers with involvement of stakeholders–local health system managers, community etc.	Planning decisions taken by NTD managers without consideration of public health system activities
	2. Priority setting			
	3. Resource allocation			
**Service delivery**	1. Structural	Intervention service delivery by public health system structures/staff or multi-purpose health workers	Service provision undertaken by public health system workers and NTD staff or service delivery linked to other public health system services	Service delivery relies on single purpose workers and have no linkages with other public health system interventions
	2. Human resources			
	3. Shared infrastructure			
	4. Operational integration			
	5. Referral and counter referral systems			
	6. Procurement			
	7. Supply chain management			
**Monitoring and Evaluation (M&E)**	1. Information technology infrastructure	M&E activities conducted by agencies/units responsible for M&E in the public health system.	M&E activities/ responsibility shared by public health system and NTD specific M&E structure	Dedicated NTD M&E structure parallel to public health system M&E structure
	2. Data collection and analysis			
**Demand generation**	1. Financial/non-financial incentives e.g. additional cash transfers	IEC activities and mechanisms to create financial incentives are provided jointly with other HS activities and undertaken by public health system staff	IEC activities and mechanisms to create financial incentives are provide jointly by NTD programme and public health system	IEC activities delivered as single purpose activities by single purpose health workers
	2. Population interventions e.g. education and promotion, social mobilization)			

Modified from Atun et al, 2010 [23].

The extent of integration of priority interventions into a health system is influenced by several other factors including the nature of the disease and intervention, the adoption system, the health system characteristics and broad context. The nature of the disease relates to the epidemiology, severity and course of an episode of illness. For example, an acute disease such as Ebola Virus Disease with high fatality may require less integration compared to HIV which has a chronic course. The adoption system involves the health institutions such as the regional and district health administrations, hospitals and the key actors within the health system involved in implementation of the priority interventions. The characteristics of the health system involve the organization of the health system at the various levels. The political system, the economic, socio-cultural and technological situation in which the health system functions constitutes the broad health system context. However, these were not specifically studied as they were assumed to be similar for the study areas, diseases and country context.

## Methods

This was a descriptive case study design. Key persons were purposively selected based on their key roles in implementation of the NTDP activities within the health system at the national, regional and district levels. In addition extensive review of programme and health system documents related to policy guidelines, standard operating procedures and activity reports was done.

### Ethics statement

Ethical clearance for this study was obtained from the Ghana Health Service Ethics Committee. All study participants were provided with written information on the study and signed a consent form before interviewed.

### Study area

The study was conducted in two districts, Ahanta West and Nzema East, in the Western region of Ghana. Western regions was selected because NTDP activities have been conducted in all districts in the region and each district had at least 2 NTDs targeted by the NTDP. Ahanta West and Nzema East districts are among the earliest districts that started NTD interventions in Ghana. The Western region is a tropical rain forest area with coastal savannah and swamps. All 17 districts in the region are endemic for lymphatic filariasis and schistosomiasis while four districts including Nzema East are endemic for onchocerciasis. Ahanta West and Nzema East districts have had 10 and 11 rounds for MDA for lymphatic filariasis respectively. Night blood surveys conducted in the two districts in 2012 and 2011 respectively showed significant reduction in microfilaria prevalence but did not meet the threshold for stopping MDA. The Western Regional Health Administration was studied as the regional level health system while the GHS headquarters from where the NTDP national office operates was included as the national level health system.

### Study population

The study covered health staff working on NTDs at the national, regional and district levels. A manager on the NTDP and two programme officers responsible for LF, schistosomiasis and soil transmitted helminths at the national NTDP office were interviewed. A Director of Health and Disease Control Officer working on NTD control activities were interviewed at the Western Regional Health Administration and 2 study districts.

### Data collection

An in-depth interview guide was used to obtain data on the structures used to conduct NTDP activities related to elements of the health system functions (Table 1) at the three levels of the health system. The guide was pretested with a manager of the NTDP.

Secondary data was obtained by review of the NTDP five year strategic document (Master Plan for Neglected Tropical Diseases, Ghana 2013–2017), MDA reports and Annual Reports of the study districts. Other documents reviewed included GHS Annual Reports, the GHS Act, and five-year Programme of Work of the GHS. The GHS website was also searched for information on the structure of the health system.

### Data analysis

#### Measuring integration

Integration of the NTDP was determined in two stages–extent of integration of each of the six health system functions and an overall extent of integration at the three levels of the health system. The two stages were assessed at the three levels of the health system independently. Extent of integration of NTDP activities assessed within the GHS headquarters represented the national level. Similarly, extent of integration of NTD activities assessed at the Regional Health Administration (RHA) and District Health Administration (DHA) represented extent of integration at the regional and district levels respectively. The overall evaluation of the extent of integration of the NTDP at the health system levels was designated as Composite Integration Index (CII). The extent of integration of each health system function provides points of intervention where the objective is greater programme integration. On the other hand the CII allows for comparison of priority interventions within a health system or in different health systems.

Qualitative content analysis was used to analyses the data using the six health system functions and corresponding elements as predefined codes (Table 1). Themes were identified while noting contrary themes. This method was used since the study sought to assess integration of each health system function and using same to estimate extent of integration of the NTDP at health system levels. Responses for each level of the health system were arrived at by observing consensus in responses and consistency with related responses. Each element of a health system function was assigned a score of one or zero. Elements conducted by the health system was assigned a score of one otherwise was assigned a score of zero. The average score of the elements of a health system function determined extent of integration of the health system function. Average score below the first quartile (< 0.25) was classified as non-existent integration; the next two quartiles (0.25–<0.75) were classified as partial integration while average score from the third quartile or higher (≥0.75) was classified as full integration.

The extent of integration of NTD activities at the national, regional or district levels of the health system was estimated as a summation of the product of the average health function score and the weight of the respective health system function. The weight of a health system function is the proportion of its elements to the total elements of all six health system functions at the district region or national level.

CII=∑Average health function score×weight of health system function

## Results

### Extent of integration of health system functions

The interviews conducted showed that the manager at the NTDP and two programme officers for LF, soil transmitted helminths and schistosomiasis had worked at their current positions for 8, 11 and 6 years respectively. The Director of Health and the Disease Control officer interviewed at the Western RHA had been at post for 5 and 10 years respectively, however, they had worked in related capacities in the region for at least 15 years each. Both District Directors of Health for Ahanta West and Nzema East districts interviewed had been at post for at least 7 years. The Disease Control Officers for Ahanta West and Nzema East districts interviewed had been at post for 2 and 3 years respectively but had worked on disease control activities in the sub-districts before their current assignments. The Manager at the NTDP and Health Directors at the RHA and DHAs were physicians with at least a Master’s degree in public health while the rest had diplomas in disease control.

#### National level

Table 2 shows findings at the national level of the health system. The NTDP is integrated into GHS national level with ultimate responsibility for programme outcome borne by the GHS. An NTDP respondent said—*“NTDs control in Ghana is the responsibility of GHS through the MoH*, *but at the NTDP level the programme manager leads*, *working under the Head of Disease Control and Director of Public Health*.*”* Respondents at all levels of the health system re-echoed integration of reporting channels made by a national respondent: *“Registers are used to compile reports at the community level and sent through the sub-districts to district then to regional level so at every level they put together every report and finally send it to the national*.*”* Two out of three health system elements of the governance and stewardship function; accountability and reporting were conducted using public health system structures while performance management was carried out by the NTDP. Programme implementation was financed by earmarked funds mobilized by the NTDP which also allocated resources through planning and budgeting to the RHA and districts. An NTDP respondent said: *“The NTDP sources funds from donors*, *government or other parties and then makes available drugs and logistic for the Ghana Health Service or the health system to be able to work in communities*.*”* However, staff salaries and office space was funded through GHS sector budget. Needs assessment, priority setting and resource allocation elements of the planning health system function were conducted by NTDP specific structures. Under the service delivery function, structural issues, infrastructure, procurement and supply chain elements depended on public health system structures while staff were dedicated to the NTDP. A respondent at the national level indicated that: *“Drugs are cleared from the ports and sent to the Central Medical Stores*, *we send our distribution list to the Central Medical Store and they ensure the drugs get to the regions*.*”* Data collection and analysis for NTD control activities were conducted using health system structures for same while the programme had a dedicated information technology (IT) infrastructure. One of the elements of demand generation; population interventions which deals with social mobilization, health education and promotion was conducted with active participation and guidance of the Health Promotion Unit of the GHS; while incentives for staff to undertake demand generation activities were absent. Four health system functions—stewardship and governance, service delivery, monitoring and evaluation and demand generation were partially integrated while financing and planning functions showed non-existent integration (Table 2).

**Table 2:** Extent of integration of health system functions at national level of health system.

Critical Health System Functions	Health System Elements	Average score	Extent of integration
Stewardship and governance	Director of Public Health of GHS ultimately accountable	0.67	Partial
	Data reported to the Public Health Division of the GHS		
	Performance management by NTDP		
Financing	Operational activities funded through donor earmarked funds	0	Non-existent
Planning	Priorities based on NTDP baseline surveys	0	Non-existent
	NTDP assessed resource needs at all levels of GHS		
	NTDP allocated resources to the regions and districts		
Service delivery	NTDP structurally a unit of the GHS and uses GHS offices Staff are dedicated to NTDP	0.67	Partial
	Procurement by GHS procurement unitDrugs and logistics managed by Central Medical		
	Stores		
	Shared Public Health Division vehicle pool		
	Operational activities not combined with other GHS interventions		
Monitoring and evaluation	Assessment surveys conducted with Public Health Reference Laboratory	0.50	Partial
	Not share Immunochromatographic Test infrastructure with other GHS units		
Demand generation	Health Promotion Unit involved in development of IEC materials and social mobilization.	0.50	Partial
	No financial incentives to improve demand for NTD		

#### Regional level

Table 3 shows extent of integration of health system functions at the regional level. Governance and stewardship function was partially integrated at this level. The RHA received and transmitted NTDP reports through existing reporting channels and also monitored performance of NTD interventions in the region. However, the RHA conducted no activity to monitor treatment outcome. The RHA indicated that the role of the NTDP in the region was: *“To provide funds*, *supply logistics*, *monitor MDA*, *and conduct prevalence studies*.*”* Operational NTD control activities at the regional level were fully funded by earmarked funds from the NTDP as stated by a respondent: *“MDA in the region is funded mainly by earmarked funds from the NTDP but indirectly funds for health education are leveraged to educate the population on all disease conditions and interventions*.*”* The RHA did not conduct planning activities at this level of the health system. All three elements of the planning function were conducted by the NTDP and not the RHA. All elements of the service delivery function except operational integration were conducted by RHA public health unit. Staff of the unit used RHA structures and logistics including shared ICT equipment to implement NTD control activities during MDA. Staff of the Public Health Unit worked across interventions including social mobilization and health education as stated by a respondent: *“All technical officers irrespective of their focus area–Disease Control*, *Nutrition and Health Promotion Officers work on NTDP and the directors supervise*.*”* The RHA provided a representative to support the NTDP to conduct MDA impact assessment surveys in the region. At the regional level, three health system functions namely stewardship and governance, demand generation and monitoring and evaluation were partially integrated; financing and planning functions indicated non-existent integration while service delivery was fully integrated (Table 3).

**Table 3:** Extent of integration of health system functions at regional level of health system.

Critical Health System Functions	Health System Elements	Average score	Extent of integration
Stewardship and governance	NTDP accountable for NTD control in the region	0.67	Partial
	RHA monitored NTD control performance		
	Existing GHS reporting system used to report NTDs		
Financing	NTD control funded almost exclusively by earmarked funds	0	Non-existent
Planning	Priority setting and needs assessment for NTD control done in the region conducted by NTDP	0	Non-existent
	NTDP allocated resources to RHA and endemic districts		
Service delivery	Public Health unit of the RHA conducted NTD control activities	0.83	Full
	All technical staff of unit involved NTD control activities		
	No integration with other interventions		
	Regional Medical Stores managed NTD drugs and logistics		
	GHS procured drugs and logistics		
Monitoring and evaluation	NTDP conducted impact assessment surveys.	0.50	Partial
	No dedicated NTD ICT equipment at RHA.		
Demand generation	Technical staff of RHA conducted social mobilization for all interventions.	0.50	Partial
	No financial incentives for social mobilization.		

#### District level

Table 4 summarises findings in the study districts–Ahanta West and Nzema East Districts. Concerning stewardship and governance the District Health Administrations (DHAs) were accountable for control of diseases of public health importance in the districts including NTDs with the District Directors having ultimate responsibility as leaders of the District Health Management Team. The DHD was considered a decentralized unit of the GHS as stated by a district respondent: *“As a unit of the GHS in the district*, *the Director is ultimately responsible for NTDs in the district but as you know he works with a team—the Disease Control Officers*, *the Field Technicians as well as other colleagues*.*”* The districts applied reporting tools and channels for the public health system to report NTD intervention data. However the NTDP from the national level conducted performance management activities in the districts. As was seen at the regional level, funding of NTD control activities in the districts were principally through earmarked funds from the NTDP. The lack of response from higher levels of health system to budgets prepared by the DHA was breeding resignation in some cases as a respondent intimated: *“Concerning budgeting we stopped doing it after a period because when you budget you don’t get the money so we just see it as part of the general health sector challenge*.*”* However Nzema East District DHA conducted needs assessment for NTD control activities in the district for each year as it helped the DHA determine MDA financing gap which formed the basis for raising additional funds from other revenue generating units in the district. A respondent in the district stated that: *“In planning and budgeting we are able to determine the resources required for the whole MDA activities*, *identify resource short fall and then solicit for funds from the district assembly as well as the District Hospital*.*”* Ahanta West district occasionally received funding support from one NGO. Priority setting and resource allocation for NTD control activities in the districts were conducted by the NTDP. All seven elements of the service delivery function were conducted with district level structures and resources. Both districts recognized health education as an opportunity for integration in the context of limited funding. A respondent commented that: *“When you have a problem whether there is money or not sometimes changing people’s behaviour helps so as I mentioned earlier concerning malaria*, *when we are conducting health education on long lasting bed nets we talk about lymphatic filariasis as well*.*”*

**Table 4:** Extent of integration of health system function at district level of health system.

Critical Health System Function	Health System Elements			Average Score		Extent of Integration	
		Ahanta West District	Nzema East District	Ahanta West District	Nzema East District	Ahanta West District	Nzema East District
Stewardship and governance	Health Administration accountable for NTD control	√^*^	**√**	0.67	0.67	Partial	Partial
	Existing reporting system used to report NTD data	**√**	**√**				
	NTDP assessed performance of NTD interventions in the district	**√**	**√**				
Financing	NTD control funded largely by earmarked funds	**√**	**√**	0	0	Non-existent	Non-existent
Planning	Health Administration conducted NTD control needs assessment	**-** ^**#**^	√	0	0.33	Non-existent	Partial
	NTDP set priorities and allocated resources for NTD control in the district	√	√				
Service delivery	Health Administration structures and staff conducted public health interventions including NTDs	**√**	**√**	1.0	1.0	Full	Full
	NTD control integrated with other interventions	√	√				
	NTDs referred through general referral system	√	√				
	Health Administration manages NTD drugs and logistics	√	√				
	NTD drugs and logistics procured by GHS procurement unit	√	√				
Monitoring and evaluation	Health Administration ICT infrastructure was used for all public health activities including NTDs	**√**	**√**	0.50	0.50	Partial	Partial
	NTDP collected and analysed data from the district to assess impact of NTD interventions	√	√				
Demand generation	Social mobilization for NTD control interventions was conducted by technical staff of Health Administration	**√**	**√**	0.50	0.50	Partial	Partial
	No incentives for staff to enhance demand for NTD services	√	√				

Note

* √ = true statement

# - = not a true statement

The DHAs utilized their ICT equipment for all public health interventions including NTDs, however, the NTDP collected and analysed survey data from the districts to assess impact of MDAs on target diseases with less involvement of the DHAs. The DHAs conducted community sensitization and health education using community durbars, community public address systems and radio stations. Community Health Nurses, Field Technicians and Disease Control Officers who conducted social mobilization for other diseases control interventions did same for NTD intervention. In the two study districts stewardship and governance, monitoring and evaluation as well as demand generation health system functions were partially integrated while service delivery was fully integrated with financing showing non-existent integration (Table 4). However planning function was partially integrated in Nzema East District but recorded non-existent integration in Ahanta West District.

### Extent of integration of NTDP at health system levels

Estimation of CII for the national, regional and district levels of the health system is shown in Table 5. The NTDP considered its activities well integrated: *“We have a national programme and it is national in character meaning that though it started at the national level it is not a targeted programme it is well integrated into the GHS*.*”* However, district level structures expected more integration for long term sustainability: *“There is too much verticalization of the health system*, *there is the need for integration that can take care of all aspects of health care*. *You know worms re-infection is common and we don’t have the resources to provide treatment when the programme ends and we have not found a way to integrate into current health system*. *I don’t know what will happen*, *it will be difficult to say*.*”* This view reflects the extent of integration recorded. All levels of the health system; national, regional and study districts recorded partial extent of integration with CII index of 0.48–0.62. However, the extent of integration was greatest at Nzema East district (CII = 0.68) and least at the national level (CII = 0.48). Extent of integration varied in the two study districts with Nzema East recording a higher extent of integration (CII = 0.68) compared to Ahanta West (CII = 0.62).

**Table 5:** Estimation of Composite Integration Index.

	Health System Level	Critical Health System Functions						Composite Integration Index (CII)
		Stewardship and governance	Financing	Planning	Service delivery	Monitoring and evaluation	Demand generation	
**Average health system function score (A)**	National level	0.67	0	0	0.67	0.50	0.50	n/a
	Regional level	0.67	0	0	0.83	0.50	0.50	
	Ahanta West District	0.67	0	0	1.0	0.50	0.50	
	Nzema East District	0.67	0	0.33	1.0	0.50	0.50	
**Health functi on weight (B)**	National level	3/17	1/17	3/17	6/17	2/17	2/17	
	Regional level	3/17	1/17	3/17	6/17	2/17	2/17	
	Ahanta West District	3/18	1/18	3/18	7/18	2/18	2/18	
	Nzema East District	3/18	1/18	3/18	7/18	2/18	2/18	
(A)*(B)	National	0.12	0	0	0.24	0.06	0.06	**0.48**
	Regional	0.12	0	0	0.29	0.06	0.06	**0.53**
	Ahanta West District	0.11	0	0	0.39	0.06	0.06	**0.62**
	Nzema East District	0.11	0	0.06	0.39	0.06	0.06	**0.68**

n/a – not applicable

### Discussion

We found that the level of integration of the NTDP was much higher at the district level compared to the national and regional levels. This was because the District Health Management team (DHMT) operated an integrated unit coordinated by the District Director of Health Service. Findings of partial integration of NTDP at all levels of the health system albeit to varying extents emphasized that extremes of the integration spectrum–fully integrated and fully targeted were the exception; rather most targeted programmes may be integrated to different extents at the various levels of the health system based on several factors [23, 33]. The greater extent of integration of NTDP at district level compared to the national and regional levels may be due to districts’ functioning as the basic service implementation unit of the health system in Ghana. The districts as implementers also felt more accountable for outcome of NTD interventions in the district. The regional level is made up of the RHA and the regional hospital. The RHA served an administrative, supervisory and coordinating role over the districts in the region. The RHA was not directly involved in service delivery. However the regional hospital’s participation in MDA was limited to management of referred cases of severe adverse event following MDA. The national level similarly has policy, planning, monitoring and evaluation roles with little direct service delivery function. This accounts for partial level of integration at the regional and national levels compared to full level of integration of the service delivery function in the two study districts. Planning of NTDP activities which consists of needs assessment, priority setting and resource allocation is centralized at the NTDP office leaving little planning at lower levels of the health system. This also means that the Policy Planning Monitoring and Evaluation (PPME) division of the GHS does not perform planning functions for the NTDP. However, Nzema East District budgeted for NTD control activities to provide evidence of funding gap and to source funds and logistics support from other decentralized departments and district local government to meet the gap. This is an important finding indicating the importance of sub-unit planning. The operational activity of the NTDP is almost exclusively funded by earmarked donor support at all levels of the health system hence the non-existent level of integration of financing health system function recorded at national, regional and district levels of the health system. Although GHS employs and pays salaries of all staff along the chain of NTD control from the national to the district level, the framework used only included operational cost and additional incentives and not salaries [23]. The need for timely reporting, and measurement of outcome and impact indicators in donor funded programmes leads to the setting up of parallel monitoring and evaluation systems. Though this was the case in the study, the limited capacity of districts in monitoring and evaluation of programmes was another reason for the national level conducting most impact assessments.

Stewardship and governance function was partially integrated at all levels of the health system. It recorded the second highest extent of integration (0.67) consistently at all levels of the health system indicating some level of leadership responsibility at all levels of the health system. This finding supports the partial decentralized nature of the health system following health system reforms in Ghana [38]. Civil society involvement in other targeted interventions such as HIV and TB has been cited for promoting fragmentation rather than integration [34]. The NTDP in Ghana does not have significant civil society involvement.

Several factors may influence the extent of integration of targeted interventions—including capacity and reach of the health system, decentralization, duration of intervention implementation, epidemiology of the disease among others [33, 34]. Health systems with high capacity and reach promote integration. The health system in Ghana has units in all districts and some sub-districts. The study sites had active RHA and DHA with sub-districts units and extension of health services to communities through services of Community Health Nurses and volunteers. The high capacity and reach of the health system in the study districts may have contributed to the high CII recorded. Each of the study districts were created over a decade ago and therefore had established administrative and service structures to the sub-district and community levels. The CII might be less in districts created more recently since it takes time to establish functioning health system structures. Diseases that have engaged public health attention for a long time such as malaria and tuberculosis have been found to be more integrated than HIV which is relatively a recent public health concern [35]. Though NTDs have been around for a long time it did not receive public attention until recently hence the extent of integration of NTDP may not be comparable to those of malaria and TB. However, the two study districts have been conducting MDA for at least 10 years each. Diseases with low epidemic potential are more likely to be integrated [34, 35]. Though NTDs have very low epidemic potential the long neglect of these diseases over the years means that consistent interventions has been lacking hence adversely affecting the extent of integration.

The extent of integration was partial at all levels of the health system with different CII. The conceptual framework made it possible to account for the differences in the CII through variations in the extent of integration of health system functions. Additionally, the conceptual framework accounted for all aspects of the programme and health system at the national, regional and district levels because it was based on the widely accepted functions or components of the health system. The study provides an empirical measure of the extent of integration of the NTDP at various levels of the health system by estimating CII. It also provides further details by measuring extent of integration of six health system functions. Serial measurement of extent of integration can be used to monitor progress where the goal is better integration. The extent of integration of health system functions provide specific intervention points to improve integration rather than targeting the whole health system. Therefore the study has application where further integration is needed as in very low disease prevalence and post elimination surveillance as well as withdrawal of donor funding for targeted interventions. In the first case, expenditure on parallel control structures may be inefficient considering the low prevalence. In the case of donors withdrawing funding, government must either raise the resources or integrate the programme into the health system for sustainability. The study can offer guidance to integrate priority interventions at a more rapid and predictable pace.

The study has two basic limitations. First it can be employed to measure the status of integration of a priority intervention in a health system but the results may not be comparable to the findings in other health systems since contextual issues such as the capacity and reach of a health system, disease epidemiology, duration of intervention and degree of decentralization are unlikely to be the same in any two health systems unless these factors can be standardized. The second limitation of the study follows from the first. The findings cannot be generalized for NTDP in other health systems since the context may be very varied.

## Supporting Information

S1 Text Transcripts of all interviews. (DOCX)
