# Supplementary material for: Extent of Integration of Priority Interventions into General Health Systems: A Case Study of Neglected Tropical Diseases Programme in the Western Region of Ghana
Source: PLoS Negl Trop Dis. 2016 May 20;10(5):e0004725. doi: 10.1371/journal.pntd.0004725 (PMC4874689; doi:10.1371/journal.pntd.0004725)
Supplement: S1 Text — (DOCX) [file pntd.0004725.s001.docx]

**Interview One**

N: Good morning again, my name is Nana Yaw Abankwah, a research assistant from the school of public health University of Ghana. I will be conducting this interview on behalf of Ernest Mensah, a PhD student from the school of public health university of Ghana. He is conducting a study as part of the requirement for this course. Thank you for accepting to be interview for the study we are grateful for your time. Indeed your input as the **desk officer for LF for the NTD** program is very important for the study. Your responses can be as brief as possible but please where you consider appropriate to do so, Ernest Mensah may come up with a few follow up questions where necessary. The interview is expected to last for about an hour and half at the most and it will be recoded and transcribed later for analysis, please do you have any comment or questions before we begin?

R: No

N: Thank you once again for your time, as Ernest said the questionnaire has been put into sections and the first part is on neglected tropical diseases the situation In Ghana. So sir my first question to you is, is LF a priority disease in Ghana?

R: Yes for now let me say so because if you look at the previous study that done the prevalence ranges from about 10% to about 40 percent in the most affected areas and even in some communities that we survey virtually everybody that we surveyed had the worm in the blood before the program actually started out so it is a priority disease that has to be tackled, it is a disease of poverty and affects people giving all them all risk from social to economic so it is important.

N: So is LF endemic in Ghana?

R: Yes once the mapping has been done that determined that district is endemic we cannot say more than that?

N: So general what interventions are implemented in Ghana to control or manage LF?

R: We have two main objectives one is to break transmission of the disease and that we do by mass drug administration annually using ivermectin albendazole in the endemic areas and second objective is to look at the morbidity aspect, manage the effect of the morbidity and do that first by hydrocele surgery by those with hydrocele and lymphoedema management for those with lymphoedema

N: Which of them was initiated by the NTD program?

R: All

N: What are the general objective or expected outcome for mass drug administration in Ghana?

R: The general objective is to interrupt transmission as I said and by that we mean that prevalence should fall below one percent and at that level it is believe that the disease would be no longer transmissible so that is what we ate aiming at.

N: So what are the time lines in achieving these objectives?

R: Our main objective is to eliminate LF as a problem by 2020 but we may be able to get there before that time looking at trend of the prevalence that has determine recent research that has been undertaken. every year we conduct there are mass drug administration in the endemic area and ensure that we get the maximum cooperation of the communities to ensure that the coverage are high, our main objectives is to achieve 80% therapeutic coverage or even higher but anything 60% and 65% is acceptable

N: So sir what timelines do you set to achieve this 80% and 65%

R: Am not clear about it, is something we set out every year

N: So it is something set out every year so you do it very year not three years, four years, and five years

R: We want to achieve coverage of 80% every year when we conduct mass drug administration and that will help us to sustain the micro filarial prevalence level and then eventually eliminate them.

**Section B**

N:So then I can say on yearly bases you set your time lines to achieve your objectives. What is the role of the NTD program in the control of LF in Ghana?

R: First the NTD program houses the LF elimination program that is to say that all that has to do with program with LF elimination is plan and implemented by NTD program through the Ghana health service using the existing structures so for example at the national level we work through the public health division through the regional directors to the regions to the district directors to the district and the sub district for activities to be implemented.

N: When you say you work through them can you be a little bit elaborate on that?

R: By that we mean that we us a coordinating body at the headquarters do not implement programs but the activities are owned by the regional directors and they ensure that the district directors also go by what they have to do and ensuring that objectives are achieved however we provided a supervisory and supporting roles in all this areas and also mobilize resources to support them as well.

N: Which officer or officers is ultimately responsible for the control of LF in Ghana

R: At the community level we depend solely on community volunteers and at the sub district level work through the community health nurses, disease control offices and public health nurse who are constantly in touch with the community and then this team is led by the sub district l health leader and at the district level we work through the district director who ensure that the subdistrict gets that their logistics and the support that they require and at the regional level we through the regional health directorate.

N: So what about the national which officer or offices is ultimately responsible for the control of LF in Ghana?

R: I will say the NTD program offices buy it is a division under the disease control unit of the public health division so we collaborate with other program areas as well.

N: So when we come to the NTD program here as in the setting which officer or offices is solely responsible for the control of LF?

R: I would not say sole responsible because we work as a team however am the desk officer for LF?

N: So I can say that at this level the desk officer for LF is Mr Samuel Odoom and is responsible for LF.

R: Yes, but responsible for LF but I assist the programs manager in planning and implementing program with the other team members.

N: What responsibility if any do the regional NTD coordinator and district disease control officers of LF endemic areas have in the control of these diseases.

R: They act as contact person for the regional health directorate, if you talk about the regional coordinators and for the district coordinator act in the capacity for the district director .Now what they do is to ensure that logistic and resources for the program are use as such. For example when at the national level we ensure that drugs and materials get to the regional level they are well distributed to the district and when it comes to reporting they also report through them to us. So they act as link through between the program at the national level and the district level where most of the implementation activities takes and they also supervise in whatever they do in relation to the program

N: What about the district officer?

R: The district officer level act similarly in the place as the district coordinator at the district level.

N: What are the reporting channel of LF epidemiological status and control activities for affected population to the national level in Ghana?

R: The reporting channels we use the existing reporting channels that are from the sub district to the district to the region and to the national level and if there is the need for a feedback it is vice versa.

N: Which officers are ultimately responsible for reporting on LF at the district at the regional and at the national level?

R: Let me start from the sub district, the sub district heads collate the report that they receive from the drugs distributors to the district level and at the district level the district coordinator who works as a representative of the district director put all this reports together and forward it to the regional focal person who also and then acts in place of the regional director and then there regional person forwards the information to the program manager who intend forward it to me but sometimes the information gets to me before I forward it to the program manager.

N: What is the role again of the NTD program in ensuring the achievement of LF control objectives?

R: First and foremost we are engage in resource mobilization making sure that there is funding there are drugs and material that we will use for the program and we also ensure that program activities kick off so for example when there is a need for mass drug administration we alert the regionals that this are the activities is about time to take place so this are the support material or logistic we are about to send to your region to ensure the program kick off and then also we conduct monitoring and evaluation activities basically we do main blood survey and we conduct transmission assessment survey and then also coverage validation to ensure that the things that are coming are genuine or are accurate.

**Section C: Planning**

N: What is the role of NTD program in accessing the resources needed to conduct LF control activities in Ghana?

R: Let me say that if you talk about resources the donor then NTD program is in touch with donor communities to supply us with drugs and some funding but we have also entered into an agreement with USAID that provides us with our main funding. With the CNTD center at Liverpool they provide us with some funding for administrative as well as M and E work.

N: I think I take the question again. What is the role of NTD program in accessing the resources needed to conduct LF control activities in Ghana?

R: Ok, Basically we have done a mapping and base on the map we know the endemic areas and we plan with endemic population of the district that are affected and looking for resources and also distributing them to the areas that require them.

N: So what is the role of the regional NTD coordinators and district disease control officers of LF endemic areas in accessing resource needs for the control of LF?

R: They provide us with population data base. Quite often we provide supplies to the regions base on the national population data and all that there are occasions they come in to say that base on A, B and C we think that the resources send to our region is inadequate so we require more.

N: What is the role of NTD program in setting priorities for LF control activities in the country?

R: The NTD program meets as a team and we set our priorities for the year and then we follow it up and make sure they are implemented so the plans are drawn by the team and share with our donor partners to be able to implement our programs.

N: What is the role of regional NTD coordinators and district disease control officers in LF endemic areas in also setting the priorities for LF control activities in the region and the district what role do they play in setting priorities?

R: Much of them are done in the national level so they do very little in that direction.

N: So in that direction they do very little or they have no role at all.

R: Yes

N: How are resources for LF control activities allocated from the national level to the regions and to the district?

R: First we have certain parameter that we use basically the population and the geographic size of the endemic areas and base on that we do the allocation and we channel it through the regional directors but before it get to them it has to be authenticated by the director of public before the resources get to the regional director.

N: What is the role of the regional NTD coordinators and disease control officers in LF endemic areas in allocation of resources for LF control activities in the region and district?

R: Quite often we do the resource allocation for our level but when it gets to the regional level they quite often make some modification depending on their needs to make sure that resources are fairly distributed. For example new districts have been created but at our level we may not have the population data and the knowledge of the geographical size of the area to be able to make fair allocation of resources but when it get to them they are make closer to the districts so they are in the better position to do that.

**Section D: Service Delivery**

N: What category of NTD program staff work on LF activities besides you?

R: Of courses the program manager and its assistant and the other NTD program staff and when plan service for example we work together as a team to be able to supervise the work on the field and all that. And then we have a team of laboratory personnel who are part of the national public health reference lab they all work together with us to be able to work on our field activities.

N: Who the other NTD staff?

R: We have technical officer and biologist supporting the program

N: Which organization or agency employs you?

R: Ghana health services

N: At your current position as NTD program desk officer for LF are you dedicated to LF control activities or work on other Ghana health services activities but unrelated to LF?

R: very dedicated but not only LF activities but when the ministry of the services decides that I should support in other areas I do it because they have been occasion where the public heath directorate plans activities in which I have been part of.

N: Do you share office space, office equipment, storage facilities, and vehicles with other staff of the GHS?

R: Yes, I am saying this because at the headquarters level we have the NTD office but the moment you move out of the headquarter level the regional and the district focal person are routine GHS staff who implement other program in addition to the NTD programs.

E: This question is in reference to you whether you share office space, office equipment, storage facilities, and vehicles with other staff of the GHS?

R: Yes, there have been occasion where other program would require vehicle in time where our vehicles are not in use but the transport officer make sure that the vehicles are in use and program manager seems not to have a problem with that, and in terms of office space, yes I work with other program staff who are staff of the NTD program officers who are staff of the GHS.

N: Do you work with other program, agency or units in the GHS or outside of GHS for the control of LF?

R: Yes

N: What are this other programs?

R: We sometimes work with the shared program because at the community level a number of volunteers are teachers and they do the drug distribution for us and bring the report to us and some are agric extension officers they work together and bring the report to us

N: So what about agencies?

R: these are the main that I have already mentioned

N: What are the essential logistic and drugs require for LF activities at the national level. Let’s starts with logistics?

R: In terms of logistic because we have to move around we need vehicles to move around then we need an office space with furniture and other things so that we can stay in one place and do other things then we require drugs for the LF we require ivermectin and albendazole for the endemic population and spontaneous to there has been a demand for managing the Lymphoedema cases at the headquarters for that matter we require drugs to manage the cases as well and even an office space to manage them there. I think there is one other major role inform by the regional focal person that we overlook and that in terms of our M and E activities they act as liaison officers between the headquarters and the region so that the district can prepare and the communities are well inform for our activities.

N: What role do you play in terms of procurement of drugs and logistic of LF elimination activities?

R: I prepare the request form and give it to the program manager to go through and submit.

E: So if you don’t procure but request what role do you play for the request in getting these drugs.

N: What is the role of the NTD program in the storage, transportation and delivery of LF drugs and logistic to the target population?

R: We basically ensure that the drugs are delivered to the central medical stores but at that level we ensure that a distribution list is prepared to enable the stores to send them to the appropriate region in the require quantity at the appropriate time and we also ensure that once that is done reimbursement to off load expenses made in charting the logistic not only drugs but sometimes IE&E materials or other logistics.

**Section F: M & E**

N: What monitoring evaluation activities are conducted by the NTD program to access achievement of LF intervention objectives?

R: First we do night blood surveys and in this we access districts that have coverage’s that are 65% or above consistently over a five years period and then we sample 500 people from the two sub district within the district to give us an idea of the micro filarial prevalence once the prevalence goes below one percent we also plans for transmission assessment and that basically looks at new infections in children age between six and seven years.

N: What ICT infrastructure computers, internet access, web site telephones is available for the NTD program for processing, storing and communication LF related information?

R: From the district level all facility own by the Ghana health service is use by the program and it is the same as the regional level but at the national level because we have our own offices, this information come to the program office directly but however we are working out systems where we would use the national DHIMS a least to store our information at the headquarters level

N: Which ICT infrastructure computers, internet access, and web site telephones do you share with other units or program?

R: At the headquarters level because it is an office and we are the only occupant of this office we don’t share this resources with any other person but once you move out of the headquarters level all resources are shared.

N: What is your role in LF M and E activities?

R: Planning, reporting, supervision and implementation

N: What is your role in data collection for LF M and E activities?

R: I am a technical officer disease control and by my training am capable of even picking blood and then preparing it and reading so am able to collect them and read them under the microscope .Am also able to organize the data to give meaning for example once you do the survey you should be able calculate the prevalence level you should be able to calculate the micro filarial density of the site and all and also report to my unit supervisor.

E: Please sir the issue of having the capability is one so do you actually do it?

R: Yes

N: What about your role in the analysis of the data?

R: Yes, of course when you are writing report on that you cannot do it without the analysis so am involve in the analysis to be able to tell that this district has pass for the next level of assessment so am keenly involve.

**Section E: Demand Generation**

N: What activities does the NTD program take at the national, regional and district level to improve coverage and increase demand for LF intervention?

R: Advocacy and the public education and we employ the use of television, the radio and all to be to get the people involved but at the district and the sub district they go further to organize community durbars and also use communities that have local FM stations to educate the people on that. One to one education with the people and even at the health facility level they take the opportunity to educate people and also use social groups also as a means to reach to many people as they could.

N: What is the nature of financial incentive available to you to improve coverage of LF intervention?

R: As a program staffs are employed by the GHS we are paid by the government of Ghana but there are no real incentive package for of us working however all our activities are budgeted for and funded as such.

N: What are the sources of financial incentive for participating in LF related actives?

R: Like I said earlier on we have partners who support us we have USAID, Liverpool that provide us with financial support and let me also not forget about government of Ghana for at least they pay my salary at the end of the month.

N: What is the nature of financial incentive for non NTD staff participating in LF control activities?

R: For example the community volunteers who work at the community level we make sure when we are planning we have a budget line to support them to do the work for us.

N: Does the NTD program conduct LF health education or social mobilization activities at the national level?

R: Yes we do and quite often I have been on TV and radio to educate the public and not just on GTV but other station as well.

N: What is your role in developing materials for LF health education or social mobilization activities?

R: Am not an expert in that direction but however when this material come up I am able to comment on that because of having work with program for some time.

N: So do help in developing the material?

R: Yes I do.

N: What is your role in conducting LF education or social mobilization activities?

R: I earlier on said that quite often am assign to speak on the national television and then radio stations as well so I present the issues as well then for those who are not technical person that have speak in other dialect rather than the official language we have to do some orientation to be able to send the right messages to the people so we have to do orientation.

N: Are you involved in developing materials for health education or social mobilization activities for other intervention other than LF.

R: Yes the NTD program does not only look at LF but other diseases as well.

N: What is the role of the Health Promotion Unit of the GHS of development of materials for LF health education or social mobilization activities?

R: Have not seen much in that direction.

E: Ok in developing the materials for health education or social mobilization has there been incidence where Health Promotion Unit has been brought in to help and develop the material of LF or is part of even conducting the social mobilization health promotion unit of the GHS.

R: At the district and regional level sometimes they are actively involve but at the national level although we use them sometimes the involvement is not all that much.

N: What is the role of the health promotion unit of the GHS in the conduct of LF health education and social mobilization activities?

R: We get them involve to get the radio stations arrange for times and personnel to speak to be able to speak to issues even in the various languages.

**Section F: We are rounding up**

N: How long have you been the desk officer for LF for the NTD program?

R: For the past eleven years.

N: What was your previous appointment prior to becoming the NTD LF desk officer for LF?

R: I was the regional NTD coordinator for the central region.

N: Thank you very much for your time and your valuable responses. We are grateful, do you have anything or comment on before we end

R: No, but you if prompt me on anything then I may speak to you.

N: No sir that would be your final.

**Interview Two**

N: Good morning again, my name is Nana yaw Abankwah, a research assistant from the school of public health University of Ghana. I will be conducting an interview on behalf of Ernest Mensah, a PhD student from the school of public health university of Ghana. He is conducting a study as part of the requirement for his course. Thank you for accepting to be interview for the study we are grateful for your time. Indeed your input as the NTD program manager is very important for the study. Your responses can be as brief as possible but please elaborate where you consider appropriate to do so, Ernest Mensah may come up with a few follow up questions where necessary. The interview is expected to last for about an hour and half at the most and it will be recoded and transcribed later for analysis, please do you have any comment or questions before we begin?

R: No

N: Alright thank you very much sir for your time. So we will start with section A as Ernest said and it is on the neglected tropical disease situation in Ghana. Now my first question to you is are NTD among the priority diseases in Ghana?

**R: let me start by saying previously it could barely be seen as one on of the priority diseases but over the at least past five years I think the priorities that the NTD are receiving in Ghana has improved.**

N: Has improved!

**R: Yes, in comparison to what it used to be, that is what I can say. So I think that gradually it is been prioritize as one of the major public health program that are been implemented.**

N: Ok alright sir. On that score what are the main NTD that are reported in Ghana?

R: What do you mean by reported? Ok mainly the ones that we see in Ghana. In terms of neglected tropical diseases I know globally there are about seventeen of them but when we come to Ghana the ones that are endemic or the ones that might be found in Ghana are about ten. Ok that we have here that people are aware in Ghana is endemic for are about ten, five of them belongs to the ones that we use mass drug administration as the strategy for control, the other five are the specific diseases where we use what we call case search and management for their control or intensive disease management.

N: Sir if you don’t mind can you elaborate on that and give me the diseases the main diseases?

R: For the ones that use mass drug administration or the preventive chemotherapy strategy those ones are lymphatic filariasis, Onchocercisis, schistosomiasis, Soil transmitted helmeths which are caused by few more parasite when we talk about soil transmitted helmet is not just one parasites the other ones that causes it there are a few other and the last one is trachoma which is cause by a bacteria.

N: Are these in any kind of order?

R: The way I named them?

N: Yes

R: No

N: Sir and the other five that are not in the MDA categories

R: We could talk about Buruli ulcer, yaws, leprosy, human African trypanosomiasis and leishmaniasis. I can’t remember any other one

N: For the purposes of this study we are really concentrating on the first five thus the one that uses the mass drug administration. Which of the NTD are considered endemic in Ghana?

R: Talking about this five?

N: Yes

R: All this five are.

N: All this five are, Ok. And generally what interventions are in place to control or manage this five?

R: The main strategies as I have mentioned earlier on is preventive chemotherapy or mass drug administration that are used for control of the five diseases that is the main strategy. If you want to prevent or control or eliminate or if you want to eradicate any of these and I forgot to add guinea worm thus very important to the other five thus the preventive chemotherapy because I was talking about eradication we must add it to the eradication program in so other to do that we use mass drug to break the transmission of any of these five diseases we are talking about. There are other control strategies that might be necessary, vector control. Significantly we might want to bring in vector control thus that is important in LF, oncho, sichtosomiasis not so much for sore transmitted helmet but at least trachoma too environmental improvement is important and that target reducing the vectors involve in the transmission it is not a vector they are carriers that are involve in the transmission of trachoma so you will realize that this are the two main strategies but in other to achieve the objective for implementing and succeeding in this strategy we need to add another thing as health education that ties in with social mobilization in other to bring about behavioral change to get people to understand and even to get people to even agree to take the drugs and then behavioral change is also necessary because we need to sustain what we bring to get people to accept so this are other strategies that are equally important apart from getting people to take the drug.

N: Alright sir. Which intervention that is the two you have mentioned was initiated by the NTD program or which other intervention as the NTD program initiated in controlling or managing the NTDs. So which intervention was initiated by the NTD program?

R: The way you have put the question is such that may be I need to give you a little history of the program. Among the diseases that I have mentioned that is the preventive chemotherapy ones the first one that had a program that I am aware of is onchocerciasis that was program that was stated in 1974 by the former onchocerciasis control program it use it to be a vertical program at that time and main strategy was vector control where they targeted vector that was the larvae of the onchocerciasis various spraying they use helicopter to spray chemicals into the breeding site of the parasite. So that move on from **1974 to about 1998** when ivermectin it was not discovered yet but that was when ivermectin was introduce for the control of the onchocerciasis and again that one targeted at least the parasite within the human host. So most countries started in 2010 Ghana was one of the countries who started but between that time in 2002 when there was devolution of the onchocerciasis control program there were issues with management of the onchocerciasis control activities in Ghana, issues with management treatment was reactive with ivermectin very reactive since it did not happen consistently and so when during that time the Lf program was mapped and initiated in about **2000 and by 2000 the lf program was put in place that used ivermectin in our mother land so around 2004 after the devolution this two program was merge under one management so that is when activities of** onchocerciasis was revived once again so then treatment of the onchocerciasis communities has been a bit more consistent and then in **2006 the USAID NTD program** was initiated and identify certain countries called the **fast track countries** they identify these countries to be able demonstrate some results that could be use into implementing similar programs other countries, Ghana fortunately happened to be one of the fast five track countries that were selected for that support so actual planning, proposal writing and everything started in **2006 and implementation started in 2007.So in 2006 the Soil Transmitted Helminth program and the Schistosomiasis** **program were also added on to LF, oncho and then trachoma basically they also rely on the MDA** was also added on but trachoma was also **managed under the Institutional Care Unit** even though they had a separated program manager the program was very closely to the NTD program manager as the case was planning in putting together the programs plans and budget and the funding for most activities particularly the mass drug administration went through the NTD program so that was what happen so basically these are the disease that uses MDA this has been the strategy chronologically this is how things has worked.

N: What are the general objectives of the MDA initiated by NTD program in Ghana?

R: The general objectives as I have said is to prevent, control depending on the kind of disease. If you are talking about oncho is a control program because of the life cycle of both the adult and the macro filarial and the micro worm most of the drugs is targeted at the microfilaria worm they are able to kill them but not the macro filarial worm so basically over the period it reduces the worm load of the microfilaria worm while allowing the adult worm to dying out of the system to go through it life cycle and die out of the system. Oncho is of an average of about fifteen the adult can live for about fifteen years, so that may be difficult for programs to target elimination as against control. Lf has always been an elimination since the adult worm life for an average of about five years so it is hope after five or six years of treatment the adult worms would have died out of the system that make elimination easier to achieve as compared to onchocerciasis which require a minimum of fifteen years, in the case of Schistosomiasis those programs target the school age so that is been more of a morbidity control in other not to complicate situation of the disease in these children as they grow into adult so again for them it always been a control program even **now but now recent empirical evidence indicate that it is possible to eliminate disease like Lymphatic filariasis, onchocerciasis and Schistosomiasis so this strategies are gradually changing because it has been demonstrated in some countries trachoma is also under elimination,** Ghana for example is under the verge of eliminating trachoma is just a few communities that are still under treatment hopefully within one or two years the treatment in those communities also should stop. I hope I have answered the question?

N: What are the time lines to achieve these objectives?

R:Initially when this program are started everybody is very optimistic with the hope that for example Lf initially everybody thought LF could be eliminated within a space of five or six years but you realize that operational issues comes into play in other to eliminate Lf you need to achieve hundred percent of coverage for all eligible people and that makes up to about 80% of the total population so if you really want to achieve elimination within a shortest possible time then that should be your target and you achieve it before you would be able to eliminate Lf but in the case of most countries you will find out that that target is really very difficult to achieve if you take Ghana for example our coverage nationally has range from 70% to 75% and recently we have something like 60,76.4 or something and in the year we actually initiated the integrated NTD program thus was in 2007 our coverage actually fell from 70% to 69.3% or something like that so then you realize that operational issues make it impossible to achieve the desire coverage so you end up treating for more years than you might have anticipated at the onset of your program so currently we have treated for about 12years. In determining you cannot stop treatment you need to come to the endpoint you need some evidence to demonstrate that you have reach to the end point of your treatment and at the time this program was started how to do that was not very clear but with time with the operation listed out this things have become clearer with the development of guideline to help country programs to make that determination so always have resulted in the program kind staggering on and taking on the various level.

**Section B: stewardship and governance**

N: The next one would be stewardship and governance. What is the role of NTD program in the control of LF, Schistosomiasis, and transmitted helminthes in Ghana?

R: The NTD program structurally in other to understand this role I need to explain. We have a national program and it is national in character that meaning that it those not started at the national level it is not a vertical program it is well integrated into the Ghana health service system at the national level. We have the program management team that manages the program for the whole country. But below the national level the same people who manage all other program been public health, or sometimes related to other institutional they are same people who runs the NTD program at the regional level at the district level it tend to be the public health or disease control team, sub district it is the clinical team because it has been merge with the public activities these people manage it for the community level so if you look at the health structure it is only at the national level where we have people who been committed to the management of the NTD program so if you are looking at the role of the NTD program it is to source funding, write plans and budget to be able to source funding for the program either from government or other parties or donors and then make available drugs and logistic for the Ghana health service or the health system to be able to work in communities to ensure that this drugs are distributed in within the community.

N: Are there any organization, programs or units in the Ghana health service with partial or full responsibility for the control LF, Schistosomiasis, and STH in Ghana besides the NTD program?

R: No, even though there are a few NGOs who have been to places that we don’t even know we only hear of them in the newspapers most of them are into deworming all the others which if you are talking about oncho have never hear of any NGO or any other organization who does oncho or LF work or anything else.

N: Which organization or program do you consider is ultimately responsible for the control of LF, Schistosomiasis, and STH in Ghana?

R: It is the Ghana health service under the MOH. Am sure you are familiar with structure of the MOH and where the Ghana health service comes in.

N: Which officer or offices is ultimately responsible for the control of LF, Schistosomiasis, and STH in Ghana?

R: At the national level it is the program manager and the routine of technical officer that assist him. And let me also add that we basically work under the head of disease control and director of public health who are also directly involved.

N: What responsibility if any do the regional and district health administrators, all administrations or directors of LF, Schistosomiasis , and STH endemic area have in the control of these disease?

R: We have some things that we expect that they would do but from experience we know what they do they are also expected to plan budget for all health activities and also implement health activities for that level but we know most of the planning are done at the national level to mobilize the resources to send to them but when it comes to actual implementation at that level they all get involve.

N: What are the reporting channels for LF, Schistosomiasis, and STH epidemiological status and control activities in Ghana for the affected population to the national level?

R: The reporting channels are when the activities get done, we work through community drug distributors at community level so when activities get done, we have people who can read and write so they use the registers to compile reports at the community level and send to the sub district to district, regions so at every level they put together every report and finally send it to national.

N: Which officer is ultimately responsible for reporting on LF, Schistosomiasis, and STH at the District, regional and at the national level? So we will start with the District level?

R: At the district level it is the disease control officer who work with the district director of health service who make sure everything is done and at the regional level it is the same or there might be an NTD coordinator at the regional level working on behalf of the regional director let’s say deputy director of public health who makes sure that all this things are done and report upon. I will was going add that because we are talking about Schistosomiasis and STH then it involve the Ghana education service or the school health education program so then we also have SHEP coordinators as we call them within that system also taking part to ensure that we have that report.

N: What about the national level?

R: At the national level it is the program team it ultimately rest on the program manager but with the assistance of the rest of the team.

N: What is the role of the NTD program in ensuring the achievement of LF, Schistosomiasis, and STH control objectives?

R: Basically the role is actually to plan, seek resources, to ensure that activities are implemented and also to report to government, other partners and other stake holders of the NTD programs.

N: Are there any organization or programs with partial or full responsibility for the achievement of LF, Schistosomiasis, and STH objective control in Ghana besides the NTD program?

R: At the moment we work with, family health international, FHI 360, USAID, non-partisan donations we also in other to improve the operational delivery we are also involve in operational implementation research and with the assistance of some support center are African LF Support Center, Neglecting Tropical Diseases Centre, Liverpool *ashanka* all these people are stakeholder and are interested in ensuring that all these programs are in place.

N: If you can elaborate a little what are some of the roles these organization play

R: Some of the roles includes making funds available in terms of financial funding the program putting money to the program others ensure that we actually get the logistic that we need such as the drugs that are distributed some of the funding goes into development and production of some material and other that most of it is funding also we go into operational research which result things to be useful.

N: What are the roles in any of regional and district health administrations of LF, Schistosomiasis, and STH endemic areas in ensuring the achievement of control of these diseases?

R: Their role is actually to plan, budget, seek resources and implement the programs and report on it as well and we know that particular when it comes to resource mobilization they don’t actively get involve in that but when we get to issues of implementation and reporting they do some planning to use the resources that are made available for them and then they report.

N: That is for the regional and the district too?

R: They do the same things

**Section C: Financing**

N: What generally are the sources of funding for the NTD control actives in Ghana are they is it government, private organization, NGO etc?

R: So far we have not had much funding from private organizations most of the funding that we have had is government, Donors internationals, Donors, NGO ,multilateral ,bilateral and stuff regarding private organization recently we have had some funding from the VRA but I don’t think we can consider that as a private almost quasi government.

N: Does government funding presented as part of general funding to the Ghana health service pool budget or it is earmark NTD program funding?

R:We don’t get much from government as funding most of which has come in regularly is from the pool funding but it is very little it is just last year and maybe in 2013 there around about and we actually had year mark funding for NTD implementation.

N: Are there situation where funds from non-governmental sources for the NTD control activities could be used to support other priority intervention in the Ghana health service?

R: We have not had such situation yet because NTD get very little in terms of funding and those funding are not able to take care of the activities that we need to do yet alone to channel some to activities outside the program.

N: Are the situation where the NTD program receive funding support from other priority health intervention in the Ghana health services such as Reproductive health Tb, AIDs, malaria etc?

R: No

**Section D: Planning**

N: What is the role of the NTD program in accessing the resources needed to conduct NTD control activities in Ghana?

R: Putting in place actually during the program of work been its annual or multi one budget as well they share it with their partners as well.

N: What is the role of the regional and district health administrations of the NTD endemic area in access needs for NTD control activities in the region and district?

R: So far we have done very little. I know that a few district have been able to work some local NGO to also source some funds and other resource to help activities in those districts or sometimes some of these are not reported to us in the national level but am aware that for example Ahanta West in the past use to get some support from world vision within Ahanta west to implement LF elimination.

N: Are there any expected roles of them

R:Yes it should be part of their work they should be able plan and budget and use those document to source for funding from wherever they are the autonomous or semi-autonomous body within the Ghana health service that allow them to do that which should do.

E: The issue here is doing an assessment of the resources required to carry out NTD activities at the region or so are they part of assessing the resource needs?

R: Yes, that is what it should be, that is what is expected to do that but experience has shown us we whenever try to do that either over budgeting , delays undertaking the activities now that we are working with all the NTD we are talking about working with a minimum of 170 districts so if you are going to wait for them to delay the work it get very difficult for work to be done and most of the budget they send to us are not budge you can work with so overtime most of the work are done at the national level.

N: What is the role of the Ghana health service head office in the assessment of the resource needs of the NTD program?

R: Basically they take the needs and all that, we are part of Ghana health headquarters so we take part in the assessment of the resources and assess the resources as well.

N: What is the role NTD program in setting priorities in NTD control activities in the Ghana?

R: Again we take the lead in that.

N: What is the role of the regional and district health administration in NTD endemic areas for setting priorities for NTD control activities in the region and district?

R: They complement with the standard at national level so a lot of what the national level decides on is done in consultation in with the regions and district.

E:Are there situation where they get set prioritizes from the region for example or district that may be for this time they think we want to do LF, schisto and there is malaria and we want to decide that at this time we treat this at time this set priorities as a unit or district?

R: I think they do because one of the things you realize that it is not always we go in with our programs and we get them done within those time period we basically go into negotiate for time for implementing the NTD program with them you cannot go to any time of the year and think that your program would be implemented so they are important.

N: What is the role of the Ghana health service headquarters in setting priorities in NTD control activities for NTD control activities?

R: Again Ghana health service headquarters take a lead, negotiate for control to be carried out and monitor what goes on and helps to ensure that reports come in.

N: How are resources for NTD control activities allocated from the national level to the regions and to the district?

R: The NTD control program resources are channel through the regional directors of health services so at every point when it get there is some bit of flexibility for them to alter because they know what happens on the ground even though we send a file with budget to guide expenditure also allow some flexibility to makes some changes where seem necessary.

N: What about the district?

R: It is the same.

N: It is from the national to the region, and then the region sends it to the district.

R: Yes

N: What is the role of the regional and district health administrations in NTD endemic area in allocation of resources for NTD control activities in the regions and the district?

R: They actually receive the resources and then distribute those resources so depending on which level you are you receive the resources from the top then distribute it to the lower level.

N:What is the role of the Ghana health service headquarters in allocation of resources NTD control activities

R: Basically we are in charge of doing that.

N: When you talk about we are in charge are you talking about the NTD program or the Ghana services headquarters?

R: The point I made was that the NTD program is part of the Ghana heath service activities so we are part of the Ghana heath service headquarters so we represent the Ghana health service headquarter

**Section E: Service delivery**

N: The next set of questions would be on the service delivery. What is the staff strength and categories of staff on the NTD program so we can start on the start strength?

R: The program that we have as now we have about almost 10 people or let say nine on the program

N: Then what are the categories?

R: What kind of categories?

N: Lets say program manager, deputy etc

R: There is on program manager, there is a deputy, one biologist, one micro biologist and three biomedical scientists at the moment we have two technical officer and one entomology technician complemented by the director of public health and head of disease control.

N: Which organization or agency employs staff for the NTD program?

R: The Ghana health service.

N: Are the staff dedicated to the NTD program or work on other Ghana health service activities unrelated to the NTD program?

R: Yes, they tend to dedicate almost 100% of their time to NTD program. In the past when there are other programs like EPI they needed people help to do monitoring they have falling on the technical team for the NTD program to help to get some of these things done.

N: Do staff working on the NTD program share office space, office equipment, vehicles and storage facilities with other staff of the Ghana health service?

R: I will say yes but usually storage facilities.

N: What about vehicles?

R: In vehicles we share when there is another program that we need vehicle and we have some there. We let them have them.

N: Does the NTD work with other program or agency or units inside or outside the Ghana health services?

R: Yes it does

N: Which are some of the other programs?

R: Like I said sometimes when there are EPI they fall on some of or technical officer to support with monitoring and supervision. And then other agency likes Ghana education service.

N: What are the essential logistic and drug required for the NTD activities at this level?

R: meaning which level?

N: The national, the regional etc

R: Even though we don’t use we do the mobilization at the national level so we are talking about the logistic, drugs you know the drugs that we are talking about but in terms of logistic thinks like registers, IE&C materials these are the ones I can mention.

N: Sir we know the drugs already but can mention the essential drugs required?

R: Ivermectin, albendazole, mebendazole, praziquantel

N: What about office, furniture, office space, vehicle all these don’t the program need it at the national level?

R: We do.

N: What is the role of this units in the procurement of these drugs and logistic?

R: We meet the projection; we fill the drug application form and response to all the queries that come until they gets approve, when the drugs get sent we are inform we play a role and make sure they **are cleared from the ports and sent to the medical stores and send our distribution list from the central medical store and ensure the drugs gets into the regions?**

N: When you say you send them you send them to where?

R: We send them to the drugs donors through the WHO, they have a committee that they are set internationally review the drug application and approve once it gets approve then the drugs donor in one of the drug prominent transit Ivermectin donation project who are responsible for ivermectin and albendazole, USAID also helps to procure some the drugs that are actually bought.

N: Is the program actually involved in the procument process to get these durgs?

R: Yes

N: How?

R: All those that I initially said are part of the procurement process.

N: What is the role of the NTD program in the transportation, storage, distribution and delivery of these drugs and logistic to the target population?

R: When the drugs get into the port, we work with WHO local office to ensure that they are cleared and send to the central medical stores for storage in terms of transportation to the regions we work with the central medicals store to either use their trucks to send to the drugs to the regions or the regions send trucks to pick them up also for the distribution to regions we send some distribution list and we work with them to ensure that the distribution list is use and if there are any gaps we help them to fill those gaps also for the logistic it goes through the same process.

**Section F: M & E**

N: What monitoring and evaluation activities are conducted by NTD program access achievement of the intervention of the objectives?

R: The first one is the report that we receive let me to even start when we comes to the actual drug distribution we do monitoring and supervision to ensure that the way things are done the method or whatever that has been taught to get the drugs distributed is what has been done and when we get to the reporting we also provide them reporting format and teach them how to use the reporting format so we monitor how those report are done and send to us as well. Having received that sometimes we also do survey to validate the report that has been sent to us after all that we also monitor the impact of the drugs distribution through drug survey because we monitor the transmission of the disease of and individuals so all these are targeted act achieving a common objectives.

N: What ICT infrastructure in terms of computers, internet access, web site and telephones is available to the NTD program for processing, storing and communicating NTD related information or data?

R: We have computers, internet access and there is web site development process that we are presently under going which was challenging in the past because we did not have somebody who was devoted to make that done now we are working together as a team to make sure that is done we also have telephone access to do routine work

N: Alright sir ICT infrastructure here again computers, internet access, web site and telephone does the unit share with other units in the Ghana health service?

R: When it comes to internet access and web site I think we can say we do share with other unit because we share the same units with program so when they come and ask of internet password we cannot hold it from them we share it with them so that they can also use our wireless. With website it is the Ghana health service website that we use even when we provide our own website we will even provide a link to the Ghana health service website.

N: Do you share telephones with any other unit?

R: Not really.

N: What is the role of the NTD program in M and E activities of NTD?

R: Basically, we take the lead in conducting these activities, we do all the planning concerning that ensures all is done, we prioritize the NTD activities as well also the ones that requires funding we make sure that funds are available to undertake these NTD activities and report on them as well when this is done?

E: Does the NTD program also directly conduct some of these activities?

R: Yes particularly for the impact we try as much as possible to be the one to do with impact assessment for most of our M and E activities we try as much as possible to directly involve our self.

N: What is the role of the NTD program in term of data collection for M and E

R: We tend to be directly involve as well

N: What is the role of the NTD program in analysis of M and E data?

R: We take it up from the national level.

**Section G: Demand generation**

N: What activities does the NTD program undertake at the national, regional or district level to improve coverage and increase demand for NTD program intervention?

R: Basically social mobilization, we conduct health education to help mobilize the communities, a lot of advocacy is done, we actually have a social mobilization plan which we try to implement, the only constrain is funding and sometimes human resource constraints, otherwise we try to do the whole spectrum of what we have on our plan.

E: These are done as part of the demand generation but for the national program and headquarter which of these done or they do conduct some of those thing?

R: At the national level apart from the planning we do involve ourselves directly in conducting these like we do programs sending people to talk on radios, television program

N: What about the region and district level

R: They do similar things as and when the facilities are available in western region for example I know they use television to generate the demands for the program because it is available there, radio are the most popular among the methods.

N: What is the nature of financial incentives available to staff at the NTD program to improve coverage of the NTD intervention?

R: Apart from salaries we don’t have any other for incentives for staff participating in any NTD program.

N: So what are the sources of financial incentive for staff participating in NTD related activities?

R: Normally it is expected that people who go to the field get paid for accommodation and some money to feed basically that is so I don’t know whether you call it incentives since they don’t get anything outside their salary such as per diem or allowance for what they do.

N: What is the nature of the financial incentive for non NTD staff who participating in NTD activities?

R: If you are working within the Ghana health services then there is nothing of that nature. Since it is not there then we skip to the next question.

N: Doses the NTD program conduct health education or social mobilization activities at the national level?

R: Yes we do that.

N: Does each unit or staff of the NTD at the national level involve in developing materials and conducting health education or social mobilization activities.

R: Yes we have done that again the NTD program took the lead and we have done that with some of our NGO partners, health promotion and PR office of the Ghana health service.

N: Does program have a dedicated staff for these activities thus developing the materials

R: No, it just the technical team that takes charge of it.

N: Does the technical team develop material and conduct social mobilization intervention activities other than NTD program?

R: No

N: What is the role of the health promotion unit of the Ghana health service in development and conduct of social mobilization activities for NTD.

R: We work with them. Actually they go organize radio and television program for us and inform about the program that some of us need to be found. Also there are other that have been very successful in working with the health promotion team like the development of posters, and we also wanted bill board we were not very successful working with the health information team getting these done but getting to the media they have play a very significant role making sure we get there

**Section H: We are rounding up**

N: For how many long have you been program manager for the NTD program?

R: I think for about five years

N: What was your previous appointment prior to becoming the NTD program manager?

R: Deputy Program manager.

N: I would like to thank you for your time for all the valuable information do you have any comment or anything to add?

R: No

**Interview Three**

E: Good afternoon, my name is Ernest Mensah, a PhD student from the school of public health university of Ghana and I am conducting the study as part of my requirement for his course. Thank you for accepting to be interview for the study we are grateful for your time. Indeed your input as the desk officer for Schisto and STH for the NTD program is very important for the study. Your responses can be as brief as possible but please elaborate where you consider appropriate to do so, may come up with a few follow up questions where necessary. The interview is expected to last for about an hour and half at the most and it will be recoded and transcribed later for analysis, please do you have any comment or questions before we begin?

R: No

**Section A: NTD Situation in Ghana**

E: Is Schisto and STH among the priority diseases in Ghana?

R: I will say it is a priority disease because looking at the age group which are vulnerable to this disease I mean Schisto and STH they are mostly from the ages of five to sixteen they are those who are affected most so looking at the picture am coming from it is a priority disease which need to be controlled.

E: Sir Apart from the people it affects do you think that GHS as a whole has set this disease as a priority in the Ghana?

R: yes, because the STH program is within the umbrella of the GHS so definitely is part of the disease that are been manage in Ghana so it is a priority.

E: So is Schisto and STH considered endemic in Ghana?

R: Yes it is endemic, based on the base line surveys that have been conducted in the previous years, the prevalence that we recorded shows that the diseases are endemic

E: So generally what interventions are implemented in Ghana to control or manage Schisto and STH?

R: Right now the main intervention is mass drug administration which is carried out in every year in all school, schools because that is the place that we can capture the target group I mean from the five to fifteen years so we carry it out in schools

E: So apart from mass drug administration are there any intervention subsidiaries to the MDA that you know?

R: Ok looking at it there is a health education component but that one is not all that pronounce if you compare to the MDA which is a bit silence which is done by the school teachers not the health workers so that one is not a bit pronounce

E: So which intervention where initiated by the NTD program?

R: Basically the MDA which was initiated by the NTD program

E: So would say that health education comes as part of MDA?

R: Yes it is part of the control exercise because if administer the drug to the child and without telling him the disease can be acquired or transmitted from one person to another then you have not done anything so you to couple it with that of the MDA to make the control effective.

E: What are the general objective or expected outcome for mass drug administration for Schisto and STH in Ghana?

R: The main outcome set by the program is to treat 75% of the children annually.

E: What are the times to achieve these objectives?

R: The times are not actually defined though the MDA is ongoing we don’t have a definite time to cease the MDA

**Section B: Stewardship and governance**

E: What is the role of the NTD program in the control of Schisto and STH in Ghana?

R: The main role of the NTD is to really to make sure that funds for the exercise and also the logistic like the drugs and the materials that will accompany the drugs to the various schools are ready and also those who would administer the drugs are been trained here we trained the teachers for the work so this the main things we put in place when it comes to the MDA or control towards the STH and the Schisto disease.

E: Which officer or officers is ultimately responsible for the control of Schisto and STH in Ghana?

R: At the national office here I cannot pin point that Mr A or Mr Z is solely for this work, what we do is we receive instructions from the program manager to carry out the various activities so if there is the need for an ultimate person in charge then I will say it is the program manager for the NTD Ghana solely responsible for the activities.

E: What responsibility if any do the regional NTD coordinators and district disease control officer in the control of this disease?

R: For the role of the regional and district officers what I will say is the continuation of what is done at the national level been transferred to this level what they do is that they prepare the grounds for the control in terms of the MDA, I mean they would make sure teachers are been informed or selected for the training they will also make sure the drugs for the exercise is assembled likewise the other logistics so upon instructions from their directors they will carry out all this activities towards the control of the activities so this is what I can say.

E: What are the reporting channels of Schisto and STH epidemiological status and control activities for affected population to the national level in Ghana?

R: Let’s start from where the drugs are been distributed it is in the various schools, so we tap the initial report from the schools and from there it move up to the district where the school is located so they will collate the report from the various schools and from there they would forward to the regions and the regions also collate the report and then it move up to the national level this is the channel.

E: You keep on saying from the school they would do this so who in a particular would do this?

R: At the school level it been done with GHS and GES so when you take a particular school the one head of the school would take the report from the various classes he would collate them and give them to the circuit supervisor I mean the one in charge of the schools within the circuit and then it will go up to the district and there too there is a scheduled officer, the district SHEP coordinator, from here the district SHEP coordinator would share the report from the circuit with district disease control officers or the district focal person in charge of NTD within the GHS sector from here the GES would transport their own to regions and then from the regions they would collate and send it to the nation and when you come to the GHS they will also collate according to the district and forward it to the region and they will also forward it to the national.

E: Which officers are ultimately responsible for reporting on Schisto or STH at the district at the regional and at the national level?

R: Let me start from the GES at the district level the district SHEP coordinator would submit its report to the regional SHEP coordinator so at the regional level then the regional SHEP coordinator would then forward to the national SHEP coordinator office. When it comes to the GHS it is the district focal person for NTD collate the report within the district and forward it the regional focal person for NTD and he would forward it to the national NTD office.

E: What is the role again of the NTD program in ensuring the achievement of Schisto and STH control objectives?

R: Let me start form the national level where we plan. First of all need to apply for the drugs that would be used for a particular year alongside we dialogue with the USAID people for the funds at this level the funds and the drugs would be taken down to the regions and from the regions we make sure the drugs get to the district level and from the district level I will say it will get to the schools.During the MDA, officers from the national office would go down to the school to monitor what is going to ensure what is done is correct and also then would move down to the district as well.

**Section C: Planning**

E: What is the role of NTD program in assessing the resources needed to conduct Schisto and STH control activities in Ghana?

R: Every year we need to target that would be treated with the year so upon getting the target we would plan towards the number of drugs we need likewise the funds from there we get the target that would be treated from the regions so base on that we distributed it accordingly to the regions and at the regional level they would do the same thing and forward it to the districts.

E: So is the role of the regional NTD coordinators and district disease control officers of Schisto and STH endemic areas in accessing resource needs for the control of Schisto and STH? May be you can start with the regions and followed by the district?

R: At the regional level they would estimate the target for the various districts so upon that number from each of the district and they would march or equate the resources according to the target each of the districts so that the drugs and funds would flow in that manner so this is what the regional focal person may do, so when we get to the district they would look at their data that is the target they presented to the region so within the district they would identify the schools with their number with enrollment so with that number they would channel the resources and see how it will get to the people.

E: Do you think that the regional NTD coordinators and the district disease officers do they plan and say that at this level we need this amount of money and logistic or drugs to carry out this activities or at the district do they determine how much logistic or drugs to carry out NTD activities at their level?

R: No they don’t do that because we at the national after getting their target from the regional front we determine when it comes to funds base on the quantum that is received from donors so we determine how much should go to a region looking at their target so those in the region do not have a connection with regards to what they receive for the year because it been decide at the national level.

E: What is the role of NTD program in setting priorities for Schisto and STH control activities in the country?

R: The initial base line and mapping out pave the way for the MDA so that determines how we need to approach the MDAs annually so upon that basis we use to carry out this priority settings.

E:What is the role of regional NTD coordinators and district disease control officers in Schisto and STH endemic areas in also setting the priorities for Schisto and STH control activities in the region and the district what role do they play in setting priorities?

R: What they do is to identify the targets for the year let’s say three regions would treating in central region this year they would first give as the figure say thirty people would be treated so they first give as the figure and secondly they would identify the places for the national say we would go to district A or district B for the treatment so with figures we come in with our planning so that we distribute logistic accordingly.

E: So do they determine the at the region or district level the time that treatment would be done.

R: They would suggest but normally it does not work that way because much of the work depends on the funds and funds are usually donated so we can’t force the donors say we want to do this a particular month when the funds are not ready so normally we wait until the funds and other logistic are available for we go in for the exercise.

E: How are resources for Schisto and STH control activities allocated from the national level to the regions and to the district?

R: It depends on the target for each of the regions so the number that we have would determine how we allocate to each of the logistics to the various regions

E: When you say we need to?

R: That refers to the national office

E: What is the role of the regional NTD coordinators and disease control officers in Schisto and STH endemic areas in allocation of resources for Schisto and STH control activities in the region and district?

R: What is normally done is that after planning within the national level, the outcome would be taken down to the regionals, so the template with regional officers will be their **guide** for the allocation or distribution of the funds to the various districts within the regions

E: So they receive the funds so do they also draw plan plans to show how they would allocate from the region to the district?

R: We feed them with our spread sheet so that one would be their guide for example I have allocated 1000 to a region am not sure when it gets to the regional level they would go by the spread that come out from the national office to the regions whether they would stick to that instructions as for that one I can’t tell.

E: So when you say spread sheet, the spread sheet that contains what?

R: The spread sheet that contains the funds been allocated to the various districts, the drugs that would be given to the districts as well.

**Section D: Service delivery**

E: What category of NTD program staff work on Schisto and STH activities besides you?

R: I will say all the officers here work on the same thing. We are managing five diseases. As I have already said this activities are been determined by funds let say in January funds for LF come we all come together towards the implementation of the program and let’s say in February funds for STH and Schisto come we all joins hands to make sure things are carried out successfully so we all coordinate on all activities to make sure there is a success in all the programs

E: Which organization or agency employs you?

R: Ghana health service

E: At your current position as NTD program desk officer for Schisto and STH are you dedicated to LF control activities or work on other Ghana health services activities but unrelated to Schisto and STH?

R: Here we have five diseases STH and Schisto are only two out of the five the remaining three I can work on them.

E: Outside this five you don’t work on other programs that are not part of the NTD program?

R: No

E: Do you share office space, office equipment, storage facilities, and vehicles with other staff of the GHS?

R: Yes, because if we touch on vehicle many at time the program may be handicapped in terms of vehicles when it is time for us to go on monitoring and other activities we do go for vehicles from other sector of the service to carry out our activities.

E: What about office space do share office space with other Ghana health service staff?

R: No, because where we are is only for the NTD program

E: What about office equipment?

R: For that one also I would say No

E: What about storage equipment?

R: For storage I will say yes we share with other looking at the drugs that we share we normally store it Tema central medical store that place is not for the NTD program but it is for the general store

E: Do you work with other program, agency or units in the GHS or outside of GHS for the control of Schisto and STH?

R: Yes because every year we do collaboration with the GES because without their involvement this program cannot hold because they hold the target group so we do collaboration with them.

E: What are the essential logistic and drugs required for Schisto and STH?

R: On Schisto we use praziquantel for the control and for the STH either we use mebendazole or the albendazole for the control.

E: You have mentioned the drug are there any other logistic?

R: We have posters, flyers, and some other reading materials that help in the health promotion aspects with regards to the transmission the prevention and the control as well so we have all this materials in place.

E: What role do you play in terms of procurement of drugs and logistic of Schisto and STH elimination activities?

R: I play an indirect role by helping in the drug application because the drugs are been donated before the donor go on they want to know the quantity that we need in a year so I have to work out an estimate so through the program manager we would forward it to them for them to purchase the drugs

E: What is the role of the NTD program in the storage, transportation and delivery of Schisto and STH drugs and logistic to the target population?

R: I will start from the national point here. When the drugs arrive at the port, it is stored at the central medical stores so whiles at the central medical stores we work out the various quantities the office would allocate to the various regions. Many at times some of the regions would come here directly for the drugs and some too we need to make an arrangement for transportation to done to the various regions in terms of the drug and other logistics.

E: When you say they come, do they come to the NTD program here or to the central medical stores to pick the drugs?

R: They go to the central medical stores upon receiving the template from our end then upon further communication we direct them to the central medical store to collect the items.

E: And you also said that sometimes you arrange for transportation to get the drugs to them what do you mean by that?

R: Many at times when it is time for the distribution it may sought of be a crash of program whereby the drugs needs to get there in time looking at the distances a region may cover before getting to the central medical stores we normally assist them with timely delivery of the drugs so we arrange for a truck to take the drugs to this regions.

E: So is it the program arranges or the central medicals stores arranges to send the drug?

R: We do it together, we come together to see how best we can arrange for a truck or a vehicle to take the drugs so we work hand in hand so that the drugs are transported.

**Section E: M & E**

E: What monitoring evaluation activities are conducted by the NTD program to assess achievement of Schisto and STH intervention objectives?

R: Let me start from the national level, what we do normally when it comes to regional training of the teachers and supervisors we go there to see how the training is been conducted in terms of quality and other indicators from the regional level we trace it down to the district that one too to know how preparedness they know towards the exercise and when it comes to the actual day that is the day of distribution we also go down to the schools to see whether they are following the lay down regulations pertaining to the administration of the drugs so we are involved right from the regional, district, down to the community level we are to ensure that the right things are been done following the indicators that have been spread out with regards to the MDA.

E: You have talked about supervision at the various level is there any activity that you conduct as monitoring and evaluation to make sure that the treatment you have given is achieving he results do you conduct any studies to find out that you are achieving your results earlier on you mentioned 75% do you do anything to find out that you are achieving that?

R: What I will say is that the next level of action of the reports gets to these offices in time where the right thing has been done with regards to the report.

E: Are there any surveys that are conducted to find out whether your prevalence’s are coming down for STH and Schisto are there anything like that?

R: Apart of the baseline and the mapping that we did at the initial stage I think this year we have not done any survey to check on the prevalence whether what we recorded during the base line survey there have been an improvement

E: What ICT infrastructure computer, internet access, web site telephones is available for the NTD program for processing, storing and communication of Schisto and STH related information?

R: I will say computer, because we officers here have been given one laptop computers to track down all the activities with regard to the coverage.

E: Is there internet?

R: Yes there are internet access whereby we do receive reports from the regional and districts level

E: Does the program have website?

R: I will say No, because those that we are dealing with we do give them our personal email and they also that by giving us their so with regard website for the NTD program I will say no.

E: Are there telephone services in the offices?

R: I will say yes, formally there used to be telephone facility but now we are using our cell phones to communicate to the regions.

E: Which ICT infrastructure computers, internet access, and web site telephones do you share with other units or program?

R: I will say No,we don’t share

E: What is your role in of Schisto and STH M and E activities?

R: When it comes to collation of the data from the various district and regions. The region bring their reports individual so we at the national office have to collate all the reports from the region to make it a national data this is what we have we been doing here.

E: What is your role in data collection for Schisto and STH M and E activities?

R: Is through the normal data of collection of data from the offices, from the district and regions. I do contact them to send their reports so the try and send them through the mail or the old system.

E: So you don’t go to the point where data is generated to collect data for example you depend on officers from the regions and districts to send data to you?

R: basically when it becomes critical and we need a report from a region and we have tried several times and it is not forth coming if the need arises we go to the point of the data generation and get the report.

E: What about your role in the analysis of the data?

R: In analyzing have already said the various regions come down with their report and let say we need to get a coverage for the whole country I need to sit down to get this coverage even though I don’t go into the details using this other models I use elementary statistics to work on the coverage.

**Section F: Demand generation**

E: What activities does the NTD program take at the national, regional and district level to improve coverage and increase demand for Schisto and STH intervention?

R: At the national level every year when we participate the regional level training we lay down our intention on the administration of the drugs. The national levels also have some messages in the form of jingle in which we give to the radio stations to air it in other to get the masses to know that such program would be coming on when you go to the regional level too they do same thing by going to radio station to announce to the people, the district level too the same thing but what I have realize is when it comes to the school program most of the parents though they hear the this thing on air that school would be dosing at this period but physically they don’t really get involve so we have to look at it upon the reports we hear every year we hear that parents restrain their children from taking the drugs and digging deep into those causes you realize they were not involved in the planning at the school phase so we need to surmount that barrier to enable more of the kids to participate in this MDA.

E: What is the nature of financial incentive available to you to improve coverage of Schisto and STH intervention?

R: Apart from the salary that get the rest what get when you are going to supervise training in a region that is the only allowance that we get even that one is not enough to cater for your need it is just your per diem for the night just a peanut but the main thing is we base on our salary there is no incentive.

E: What are the sources of financial incentive for participating in Schisto and STH related actives?

R: It is part of the donor’s package from the USAID end

E: What is the nature of financial incentive for non NTD staff participating in Schisto and STH control activities?

R:Every year when budget is been prepared to the various regions the budget do cater for those who would be trained at the training section and those who would going for supervision for that short period I think apart from that there is no incentive package with regards to funds for any other staff

E: You said apart from their supervision so when they are going for their supervision are they given any incentives for the supervision?

R: Looking critically, even that one as for supervision is only the fuel that they provide because the donors assume that if you are moving in your own region it is the fuel that you need.

E: Does the NTD program conduct Schisto and STH health education or social mobilization activities at the national level?

R: We have develop a jingle and every year we activate the jingle to announce to people that such an activity would be coming on so far this what I know of in terms of health education and social mobilization

E: What is your role in developing materials for Schisto and STH health education or social mobilization activities?

R: We work in a need so when the need arises for the development of materials we all sit done together and plan towards the development of materials.

E: What is your in role conducting for Schisto and STH education or social mobilization activities?

R: Is the same response hereby here we team up it is not sole responsibility of Mr. A or Mr. B

E: When we come to you for health education and social mobilization what do you do you participate in the health education or do you go to the radio to go and talk or something.

R: For radio talk I have not done something like that but the office at large we do but for me I have not gone to the radio to do something like that

E: Are you involved in developing materials for health education or social mobilization activities for other intervention other than Schisto and STH?

R: For the other three diseases I will say yes

E: So outside the three diseases are you not involve?

R: No

E: What is the role of the health promotion unit of the GHS of development of materials for Schisto and STH LF health education or social mobilization activities?

R: We team up when the need arise for the development of this material, the jingles etc

E: What is the role of the health promotion unit of the GHS in the conduct of Schisto and STH health education and social mobilization activities?

R: They have the expertise when it comes to this field so they have been giving us the advice and they too they also much involve to see that the program or activity is conducted successfully

E: Do they take part in the actual health promotion and social mobilization activities?

R: At the national office here before can even be aired the greater Accra unit team up with us, they would draw the program and the itinerary so on that ground they do much of the coordination.

**Section G: We are rounding up**

E: How long have you been the desk officer for Schisto and STH for the NTD program?

R: I will say six years

E: What was your previous appointment prior to becoming the NTD Schisto and STH desk officer?

R: I was in upper west region as the regional focal that time it wa only the LF program that was running

E: Thank you very much for your time and your valuable responses. We are grateful, do you have anything or comment on before we end

R: My comment is if we can share after your study your findings so that we can improve what we are doing right now.

E: Thank you very much.

**Interview Four**

N: Good afternoon once again. I am Ernest Mensah and undertaking a fellowship at the University of Ghana School of Public Health and conducting this study is a part of my course work. I want to thank you very much for accepting to be part of this study and very grateful for your time and also your input as Regional NTD Coordinator is very important for the study. Your responses can be as brief as possible but please elaborate where is you think is necessary to elaborate. So the interview is expected to last at most 1 hour then it would be recorded and later we would transcript it then analyzed it. And I want to thank you, once again if you have any comments, before we start;

R: O I don’t have a comment

N: It is well taken

R: I am also willing to cooperate with you

N: Thank you very much. We have divided it into sections .First is the NTD situation in the Western Region. We would talk about that one and the way the program is managed and we would look at financing issue, look at planning issues and also look at demand generation or social mobilization, we would look at M&E , those are the categories the questions would cover. We would start with the NTD situation in the Western Region. Are NTDs among the priority diseases in the Western Region?

R: No because I may say it’s a speed program so in actual fact we depend on the natural level but as a region it is not one of our priority programs.

N: Okay, so the disease, unless I break it down. You know there are programs for Schisto; There is a program for Yaws. They have enough programs so because they don’t have resource to deal with. That why I am saying it is not a fact for endorsing because is the national that support us to undertake those activities.

N: so does the Western Region think that when you look at the diseases that are important for then in the district, are NTD one of them?

R: Yes, NTD is one of them

N: That is very important

R: Yes, they are very important disease

N: Okay, so and which of the NTD are reported in the Western Region

R: These NTD’s reported are Yaws, Filariasis, Oncho and the Schisto mostly they are not widely spread. They are transferred to locations.

N: What about Soil Transmitted Helminths STH

R: Those considered endemic are one, the Oncho, schistosomiasis , the filariasis and then the Yaws and then Antihelminthics (STH).

N : STH

R: Yes, those are the ones

N: So for this study, we would concentrate on the one that is done by the NTD programs that is the Elephantiais and then the STH and you also mentioned Schisto so the question would be around those. So generally what intervention are there in to the region to control these diseases .

R: Generally what we do, because of all these drugs what we normally do is we depend on the national level, generally they do education in to the community example, those schistosomiasis . The community members are educated not bath in the contaminated river bodies but with the oncho to we also tell the farmers to cover their bodies when go to the farm and with the filariasis too. Because is also associated with mosquito vector people are also educated on how to protect their bodies from been bitten by mosquitoes, generally our emphasis are on education rather than giving drugs . Thus the drugs are more expensive.

N: So you do education, that is one aspect and apart from education what else do you do?

R: what we do again is when we receive drugs from national level, we do yearly distribution of drugs to endemic communities.

N: That is mass drug administration.

R: Yes, mass drug administration.

N: so which of the intervention that you have mention were intervention brought by the NTD program?

R: Those brought by the NTD program are 1 oncho, filariasis and then schistosomiasis these three are the intervention given by the national level on yearly bases.

N: so, the intervention is it education on mass drug administration?

R: No they do mass drug administration as well as education because we were given leaflet to communicate. So they would be aware how the disease transmitted and then prevented so we do the two aspects together.

N: so what are the general objective or expectation of mass drugs administration in the region?

R: In the region our general is to reduce the incidence of blindness as well as prevention of filariasis and we also want to make the people take the drugs during the period is due

N: so are there time lines to achieve these objectives ?

R: Yes there are time line for oncho and filariasis. I think is 50 years, so after each five year duration they come out to do monitoring that is they want to know the endemicity of the disease among the populace because they have been taken for 5 years. so every five years they take from national and come down here to do a study to see the endemicity of the disease in those areas .

N: so you think that are these sometimes that is set aside that by this time we should get their level to eliminate?

R: Yes because they say 15 years continue supply of the drug should be able to get rid of the disease.

N: Are there any other intervention or activities which is initiated in the region to control these NTD’s that were not brought by this program?

R: Yes what we normally do I said has been the mass education to go create awareness to the FM stations to the public to tell them how the disease is spread and what to do not to spread the disease so these are what the region use to put up before the drugs came so these are some of the ways the region has taken upon itself to put in place to reduce the incidence of the disease among the populace.

**Section B: Stewardship and governance**

N: Thank you very much so we go to the next section that is stewardship and governance .so what the role of the regional health administration in the control of LF and schisto and STH

R: In the western region we have drawn up a plan which involve the community members, we want them own the program as their own so that when you put measures in place they can also cooperate with us to do those activities so the region have a plan for that.

N: so in other to control these diseases what role does the region health administration play?

R: The role RHA plays is 1 when we receive the drugs from the national level then make sure the drug is delivered to the district and also train people how to use the drug and ask to recruit volunteers to be use because they cannot do it all by themselves so these are the measures put in place as a region.

N: so what is the role of NTD program in the control of NTD’s in the Western Region?

R: The role of the NTD program is they 1. Do national assessment, so after the assessment then they give us the drugs for us to do distribution. So they have guidance for the distribution. Drugs are given to us, they train us how to train volunteers, they supply all the needed logistics that goes along with the mass drug administration. so they give us the drugs needed logistic finances was also given so take the whole lot of activities so far as this region is concern.

N: so what agency or organization and program do you consider that they are ultimately responsible for the control of NTD’s in the Western Region?

R: Agents are the national oncho filariasis program they are the main agent formerly urge supported by Sight Savers but it looks like Sight Savers has withdraw it , services so now are all depend mainly on the national program

N: so, you think that they are the main organization are responsible for the control program

R: Yes, responsible for control programs to the western region.

N: Which officer (s) is ultimately responsible for the control of LF, schistosomiasis and STH in the Western Region?

R: Officers are mostly disease control officers, they are technical people because they are in connection with the disease so they are the main people who are in control of the NTO’s in the region

N: so apart from them, for example someone comes to the region wants to hold someone responsible for the NTO’s situation in the region. Who immediate is finally held responsible?

R: The deputy director of health have all these technical people report to the deputy director of health to throw the of the NTD program focal person if it falls on the deputy director in the region before you find the focal person of the program in the region

N: so what responsibility if any do the district health administration in endemic areas have the control of disease, where the disease is endemic what role do they have?

R: The role that they play are many but the most important ones are

1.securing the drugs from national level

2.securing all members connected to the program

3. And then monitor the trends of the diseases in the region

N: so in the district what role does the district health administration look in the control of NTO’s in their district?

R: 1.They recruit because it is a map track type of program. They recruit volunteers who would support help the health officer in the distribution. One also take stock of drugs and also logistics associated with the program. And do distribution to various communities through the health officer and volunteers. So they monitor the giving of the drugs volunteers and the returns whatever is associated with the disease

N: And what are the reporting channels for the NTD control activities and status in the region, looking at it from affected community in the national level.

R: 1. When the drug comes they first given to the volunteers,

2. volunteers submit the reports to the sub-district head, the sub-district heads collate the results and send the summary report to the district head who also collates all sub-district reports and sends a summary to the region. The region send summary of the districts to the national level

N: At all these levels which officers are particularly responsible for making sure that the data or information move from the level to another.

R: The officers are you know we have appointed focal persons at various levels , so at every level there is a focal person for NTD’s at certain level the technicians are appointed as focal persons, so does the volunteers are those people collate the data to the national level that is the sub district, at the district regions, they are the disease control person which met the focal person and at the regional level; the regional disease officer met the focal person.

N: so what role does the NTD program play in ensuring that the objectives NTD program is achieved?

R: The role they play are;

1. Making sure the drugs are delivered on time

2. The officers are trained on time

3. The coordinators are also trained so that they can move and monitor the program.

Ensure there is a dead line or period for submission of reports. They should monitor all these activities and they come in person to supervise the work. Thank you very much. We move to section C that is financing.

**Section C: Financing**

N: So what generally are the sources of funding for the NTD control activities in the region?

R: In the region, the sources of funding is from the national level because the region finds it difficult to find money therefore the program manager have been depending on the donors who factor these program through the program manager

N: How is the NTD’s activities funded when it comes to GHS pooled funds in the region and then also the money we talk about from the NTD program thus these some of the poll funds try to support these a NTD’s activities apart from the earmarked activities

R: No, it is only the funds earmarked that comes to support the activities. But when we had sight savers then we had the two funds together. We use sight savers fund to pay social mobilization and we use the NTD one to pay the volunteers. Right now they have withdrawn their services so we just depend on the program funds.

N: Sight savers used to support the region directly?

R: Yes, directly

N: so are they situation where funds from the NTD program for NTD control activity could be used to support other priority interventions in the region?

R: No, when the funds is mainly for NTD’s except when it coincides with any regional activity or program then we integrate all the program together but most at times they are use for NTD program

N: So are there situations where the NTD’s control activities are supported with general regional health directorate funds from other priority disease like Reproductive health, TB, malaria are in the region.

R: No, but now because of need fund flow in the region we integrate programs. So what funds available is used to integrate with each other programs activities.

N: So it is possible or does it happen that funds from their programs or diseases we use to support NTD’s control activities.

R: For example, I think last 4 years or so when we were doing the NTDs we integrated guinea worms and with NTDs, because pictures were given to identify NTDs , at times where given to identify new uses. It is integrate , though the money were meant for NTDs but when it comes to NTD’s programs are schedule in order not to coincide with any of our programs. We just focus mainly on the NTD program because it is type bound. Thank you, we would to the next Section that is planning.

**Section D: Planning**

N: What is the role of the region health administration in assessing/determine the resources that are needed to conduct NTD control activities in the region ?

R: After the end of the each program, we collect data and ask them to forecast it at 25% so that we all contribute our input into the station aspect. We use population to forecast our tracks coverall and when that is done we collate it at the regional level and it to the national level

N: when it comes to funds that are related to the problem, do you also plan, say well this how much we need for the program.

R: For funds planning , we do all the time but most at times we come over board .Look at the number involve , and the amount. Involves cannot be borne by the national program so we a share it so it becomes equal for all volunteers thus funds are not been accurate .

N: so what is the role of the district health administration where NTD is endemic and also assessing the resources they need to control NTD activities in that district.

R: For these under big communities , it has always been difficult because they put in their requirement but national scale it down to 40-44 and national because the district do not have funds they at times supported volunteer where the funds is required of concentrate to what funds left are to plan allocated .

N: What is the role of the NTD contracted in the region?

R: In the region it has been a problem as I said, they wanted to draw the national program to access our entire requirement, and also they also seek support outside their environment so it makes it difficult to give out the actual resources. They give us what they get

N: What is the role of the regional health administration in setting up priority for NTD control activities in the region?

R: In actual fact, the regional health has also developed it’s time of programs, so what the months what I am saying is fitted into the regional program. So always there is a time for activities in other regions

N: So it means the regional estimates the time that they think national would do it/MDA and fit it in their plan?

R: Yes.

N: so the region does not determine that they want to do it at this time or another time

R: No, because when the national or program manager bring his plan and they look at it and it does coincide with the regional activities we stick to the plan that it sent to us then we readjust the plan that suit what the national wants.

N: What is the role of the district health administrator and also setting up priorities for NTD activities in the districts?

R: You see , the NTD activities have being fix around January or October so this periods are left blank waiting for the national to come out with time line for mass drug administration. So during they plan they also set some two months as outstanding so if there is any no plan, they also use it to do their normal work planning.

N: What is the role of the NTD program itself and also determining the priority for NTD activities in the region?

R: Mostly what we do is that we come to a consensus that January or October is set aside for mass drug administration so national plan with the regional level that is why if the regional was thus we hear from the national level, if the is nothing then we quickly fit in a plan program to suit those 2 months.

N: What is the role of the NTD program in resource allocation for NTD activities in the region and the district too?

R: Actually what the national level does is NTD program because they didn’t accept to them, they look at the data and our requirement, so base on the data and they supply our requirement. So requirement is given to us are base on data submit to the national level that determines our requirement.

N: What about resource allocation in the district. What role does NTD program play?

R:The role play is that base on popularities when data is prepared it is segregated district by district so therefore they know each district performance and populations. So it is projected and base on that one, our needs through that number of people treated in that particular year is projected about 5% and look at that and give us the required logistic needed to work which put extra factors at our aid in order to save wastage of loses base on our population performance.

N: What role does the district health administration play where NTD are allocated, that is the role they play in resource allocation in the region.

R: The role that they play at the region is the same as the role play at the national level. They look at the drugs use previously and add some buffer to it so that there would not be any shortage. They ensure that the drugs are supervised at endemic places but not diverted. Dealing with the sources requirement

**Section E: Service delivery**

N: So we look at the next section on service delivery. So what category staff at the regional health administration works on the NTD control at the regional level?

R: We work with the district and order intersectional bodies like teachers and the general public. Those who have influence on the programme we work with them planning and training so that they can go out.

N: At the regional health administration there are various staff here which of these staff work on NTD.

R: We work with most people on the public health background are those we work with but the community nurses, nutrition nurses and public health nurses.

N: So which agency employs this staffs who works on the NTD activities in the regional level?

R: The agencies are Ghana health service workers and some belong to MDA like when the teachers come in because of the education that we need we also include the teachers in the program so that they can go out there and propagate the importance of the drugs to the community but they are paid by their agencies.

N: So are the staffs on the NTD activities dedicated to the NTD activities or they work on other things apart from the NTD.

R: Apart from NTD, they have other things to do but when mass drug administration period is due then they all come on board.

N: As the regional NTD coordinator are you dedicated to NTD only or perform other activities in the region?

R: I perform other activities in the region such as disease surveillance and other activities assigned to me by my supervisors.

N: So do staffs at the NTD regional offices share the same offices, offices equipment, vehicles and medical stores with other staff at the administration who do not work on the NTD control activities.

R: NTD’s you can see has not got any object so it is donor pull, we all use resources together if NTD want to go up, they just borrow vehicles from other department, if we want to use computer there is a common one for use and at times I use my personal laptop to enter my data so I can have time for it, so we do not have common office.

N: Are any NTD control activities taken together with other control activities.

R: Yes, though if the mass drug is not due and they are going on outreach disease surveillance. They do sensitize the population about the NTD’s remind them when the time is getting nearer. These are integrated at the district and regional level.

N: So what referral options are there in NTD cases that are peripheral health facilities?

R: During the mass distribution few drugs are left at the health facilities levels so when the case is referred at the community level, it is referred to the health center, if they are not able to get then they are referred to the public health department where most of the drugs are kept, oh sorry few of the drugs to be used.

N: What are the essential logistics and drugs required for NTD control activities at the region.

R: Logistics required are registers, for the logistics we have a register that we register the cases or treatment or treatment we have to register everybody in the community so the registers are most essential to us and the leaflet there is an educational manual which is given to a community, they are also one of the measuring poles as well as the drugs both ivermectin and praziquantel as well as the albendazole.

N: What is the role of the regional health administration in the procurement of these drugs?

R: These drugs and logistics are mostly procured by the NTD program but we are just caretakers of these items. They are procured at the national level by NTD programs distribute to the various regions as well as district and district level.

N: So you do any procurement so far as the drugs are concerned for this program.

N: And u personally as the NTD co-coordinator for the region what roles do you play in the procurement of these drugs and logistics.

R: The role and play is to make some input, either what we need and quantity we need are the input I put up across and in terms of accessing the drugs I don’t play any part.

N: So what is the role of the regional health administration in the storage, transportation and distribution of these drugs?

R: At the national level the regional health administration assist by storing the drugs at the regional medical stores and also provide vehicle to send the drugs to the district to program co-coordinators because I do the distribution, give the request to be approved and it circulated to the various district .Then the regional health administration provide vehicles to send the drugs to a venue for them to use. So the same thing is done at the district level, the district health administration to the sub-district level. They distribute and the coordinators are done in the course of the program, they are trained and in the course of training drugs are given to them to take to their communities whiles administration is ongoing. We are going to the next section that is monitoring and evaluation.

**Section F: M & E**

N: We are going to the next section that is monitoring and evaluation. What monitoring and evaluation activities are conducted to access whether the objectives of the interventions have been achieved?

R: At the regional level, what we do is form monitoring teams and then we allocate them to the districts for 7 days whiles the program is ongoing, to see how the drugs are being distributed, how the others are given up to the volunteers or community members. So this is what goes on at the regional level. Regional director provides vehicles, formation of teams dispatch them to the various district as well as community level.

N: Apart from supervision, do you do anything like laboratory investigation to find out or survey to find out how the treatment is going on or the enderminity is reducing?

R: What we usually do whiles the program is ongoing is we monitor, how the drugs are given and how the people are measured. We also look at the way people respond to treatment because it is taught that is through direct observation when we give them the drugs but for the enderminity of the program, people have been trained at the regional level but not started on their own now. So mostly it is the national level that comes down to join the regional team in the survey or assessment of the program whether the cases are going or worms are still in people’s bodies.

N: So what I.C.T infrastructure, by this I mean computers, websites, internet access and telephones is available at the region to process, store and communicate information or data on NTD’S?

R: We have internet or data or wireless and laptops so when data is entered we send it to the district level to see their performance so more or less a feedback is given to the district and at the end of the program we do a revive so that we point out where we fall short and then build upon them next time. So this is the way we do our program.

N: What about to you have a website?

R: Western region health administration has a website on the internet but in fact I just give a copy to the public health director and regional director. I don’t know whether I have put it on the website, I can’t tell but I remember one of our training after the exercise a private journalist put the outcome on the website.

N: Which of the I.C.T infrastructure like computers, internet etc. are the unit shared with other unit or program at the regional health administration?

R: We are using dims, so everything goes into the dims so that it is shared nationwide as well as outside the country. So everyone has access to the dims

N: So computers and internets apart from NTD’s you share it with other programmes.

R: Yes we share with other programs

N: What is the role of the regional health administration in M&E activities in the NTD in the region? Here we mean of laboratory testing, survey what role the region play.

R: The laboratory test, I think they do in consultation with the national people because I understand there was a filariasis case, people from outside the country came down to support these program but the region always rely on external supporters to do certain survey but most of them have been done at the national level.

N: What is the role of regional health administration in data collection for NTD’s, MNE activities.

R: The role is that we have health information officers there so the data is passed through the information officer to regional level through the internet into the DHIMS but if it is a fresher program like Mass drug administration then they work on it so fast that it reaches the region on time, so it is transferred through email to the region. So the region sometimes sends a reminder to the district when the program has ended, that data is required.

N: What is the role of the region health administration in analysis of NTD’s and M&E data in the region?

R: The analysis has always being done by program focal person and then a feedback is given to the director of public health which is shared within review and also put into the regional annual report for them to see.

N: What is the role of NTD program in collection of data for NTD M&E activities in the region, the national program?

R: What the national does is that during the training we are given time period to submit the data so when is due then they start calling or send message through the internet to the program focal person in the district reminding him that the time is due for the data to be sent to the national level. So this is the way M&E activities is done by the national level through telephone calls and internet communication reminding the other person the importance of the data submission.

N: For example when it comes to surveys that the national program conducts, if they have to conduct any survey within the region. What role do they play in the data collection in the national survey results? What role does the NTD program itself play?

R: Usually they write to the region and the region would select by medical scientist trained, so they are trained and they all go out with the national team so the national team serves as supervisor to the regional team. From the spot data is generated and send back to national level.

N: So what is the role of the NTD program in analysis of these data at the regional level?

R: I think most of the time the analysis get outside the region because of time constrain you know they are also working on time and so they may do some but the final analysis is done at the national level and the feedback is given to the region. At times too briefing is done before they leave and then report is submit upon the activities done at the district level. So now we would be talking about demand generation

**Section G: Demand generation**

N: What activities does the regional health administration undertake at the regional and district level to improve coverage and increase demand for NTD intervention.

R: As I said earlier on as been the educational aspect of it, so that we try to sensitize the communities for them to grave for the drugs so that when he time is due they also rush. Because the program has been so synthesize they know the free periods, a times when you are there they would be calling asking has the drugs come or when is it coming, so the demand is so great because of the interaction the region has put up with the communities and the districts.

N: So what medium do you use for these mobilization activities?

R: They do community debase but at the regional level we have being using fm stations. On every Wednesday we go there to talk about most of the diseases especially when MDA is during closer we start a week earlier, educate the public, and sensitize them for the program so the same message is also sent to the district level. Those area where they have the FM we use them, those who do not have use the PA system. We go into the community to sensitize them and the teachers also are helpful in these area because they are recruited into the training, so they also go out and tell the students, the school pupils and as they go home, they go propagate the message to the parents, this is the way it’s done. And because the community officer have relies the importance of the drug and they know the deficient period and so they have been demanding when the time is getting closer, they would be calling you, so a times you also force national when are you bringing the drug, the demand at the sub-district level is so great and we keep on reminding national.

N: What is the major or financial incentive available to staff of the regional health administration to improve coverage of NTD intervention?

R: Financial aspect is not something good to write home about, why? Because always funds coming as compared to NID’s and other programs is too little but because of the love we have for the program and because we want our people to be healthy so we have volunteer to do it irrespective of inadequacy of the funds. But at the sub-district level and the volunteers that is where the problem is when it comes to allowances or financing given to them is too little so most of the people try to opt out because when they compare that one to the NID and to other programs it becomes a big problem.

N: So what are the sources of these little financial incentives that the staff who are participating here gets?

R: It is from the program, NTD program national level.

N: What is the nature of incentives for non-RHA staff participating in the NTD activities programs in the region?

R: RHA staffs participating in this program are given something small from the national level for them to do monitoring and supervising the program but then most of the money is channel to the sub-district and district level, where the activity actually goes on

N: But do you have some people who are not staff of the regional health administration who participate in the NTD activities at the regional level

R:Yes,we have some people like that because when it comes to monitoring and supervision, we try to recruit non technical staff to join the team because they are coach what to do when they are on the field and also when we are doing the regional training they are part of it. You know before the regional training we do some planning, we try to review the past experiences, try to educate them what to do. Whiles they are on the field we prepare a checklist, pay them through before they join the public health staff to do the monitoring on the field. Those going are given small per diem so that they can cater for themselves whiles on the field.

N: What are the sources of financial incentive you give to those who do not belong to the regional health administration but support the activities of the NTD’s?

R: Yes, from the same funds that come from the national level account, on the attach regional level has being able to top it small when he found out that the funds is too small. They top it up that people going benefit.

N: So does the regional health administration conduct health education and social mobilization activities at the regional level.

R: Yes he, there is a focal person on health promotion so he does educational aspect of it.

N: Which unit or staff of the regional health administration is involved in developing materials for NTD health education and social mobilization?

R: It is the health promotion but mostly they are sent at the national level and he adds something up and a time translate it in the local language, so that people would understand what is happening

N: So the materials are actually sent from the national level.

R: Yes, national level

N: Does this unit or staff develop materials for health education and social mobilization activities which is for other interventions rather than NTD’s

R: Yes, but usually we look at the program and come out with some messages from it. So sometimes it is not regular and a time the program comes with its own messages which they use it to do the education.

R: So apart from helping with NTD activities do they help with other activities.

R: Yes, they help with all other prevailing activities

N: The public health do they themselves conduct health education and social mobilization activities themselves?

R: Yes, they do .The staffs are few they are just two officers so other public health staff join them conducting the promotion activities. They develop and share the message with other high staff to take them so that they all join hands together.

**Section H: we are rounding up**

N: So how long have you been working as regional NTD coordinator?

R: There was a focal person but now he is in school so I work in conjuction with him.So I may say more than ten years.

N: So what was your previous appointment prior to becoming the regional coordinator?

R: I was the officer in charge of the disease surveillance. So when he went to school, whiles he was working I was working with him thus when he went to school he just handed over to me because I have being with him for a long time, I acquire some experience through the programs.

N: So thank you very much for the information you have given us, d o u have comments or anything to say?

R: All that I have to say is as you are doing research on this one, in the recommendation you have to impress on the people, to increase the allowance of the volunteers because they compare allowance to other programs and then the endemic areas especially mining is going on and they turn to fuel out, normally they come for training when the time they run to do galamsey or cocoa business because that one pays more than the program so I think. So I think the program needs to increase allowance a little.

**Interview Five**

N: Good morning once again

R: Good morning

N: I am Ernest Mensah, PHD student of the University of Ghana School of Public Health. And this study is part of the requirement and I want to thank you sincerely for making time from your busy schedule to meet me and also accept to be interviewed for this study. I am very grateful for your time and indeed your input as the **director of Nzema East** is very important for the study. Your responses can be as brief as possible but please elaborate where you consider it appropriate to do so and the interview is expected to last about an hour at most and it would be recorded and transcribed later for analysis. Please do you have any comments and questions before we begin?

R: I fully accept this interview and very happy to be part of this endeavor

N: Thank you very much. The question are divided into sections, the NTD situation, then we look at the management issues, service delivery, financing, planning, issues of demand generation and then monitoring and evaluation. So we would start with the first section on neglected tropical disease situation in the district. So the first question is, are NTD’s among the priority diseases in the district?

R: Yes, they are

N: Which NTD’s are reported in the district?

R:We report on Onchosiachasis, YOURS, Chisochomiasis and other enigmatic conditions and also phyliariasis so these are some of the diseases we report on relative to NTD’s.It is a priority because is a combination of the indemicity and impact on the community and everything and that is how we concern it as a priority.

N:Which of this NTD’s that you have mentioned are considered endermic in the district.

R:Onchosiachasis and chisochomiasis are endermic in the district.Phylsiasis I am not too sure.

N:Generally,what interventions are in place in the district to control these NTD’s

R:As far as onchosiachasis is concern we have distribution of drugs to communities once a year and that is a program that goes on every year.And then for chisochomiasis there is also a school health program that goes on periodically other than that those who have acute form of the diseases when they come through the routine health system they are seen and treated.So what is means is that institution stock the drugs so that they can manage the cases as and when they appear when they come to the clinic.

N:Do you also undertake health education issues in relation to these diseases.

R:We do,the community health nurses they are a group of people who routinely interact with the communities by the nature of their work,it is an outreach program.So they as part of the health education program,before they start work they do this exercises.They also make it part of their efforts during home visits.We plan to do it on air because it would give us a further reach but that has not been possible due to some constraint so as of now the means of educating communities on these NTD’s is through the community health nurses during their outreach programs

N:What are the general objectives or expected outcomes of the interventions you have mentioned.

R:First of all is to create awareness and also remind the communities about the impact of the disease and also how disabling the disease is and therefore the need to embrace the interventions we have in place in terms of community drug distribution and for those who have covet manifestation of chitosomiasis we expect that they would come to the hospital and also to avoid contact with the sources of the causative agent but as far as onchochiasis is concern it is endermic therefore we ensure that when you catch the causative agent we can deal with it,because there is no way we can break the contact between the agent and the host.So these are the objectives,first of all to create awareness and then encourage them to embrace interventions to contain the condition.

N:For the interventions particularly for the drug distribution that you undertake,do you have any objectives for undertaking them

R:Yes,first of all the objectives is to reach proportion of the target group.So we normally expect to hit 80% or more that is the objective.I think over the years we have been able to release these goal.That is the key objective to reach many people as I said 80% and that has been achieved.Ocassionally we have people reacting to the conditions and there is a system in place,before the drug administration there is wide intereaction so the system in place where they are encourage to visit the nearest health facility and they are treated free of charge.They are given preferential treatment because they are not allow to go through this whole waiting thing.So theses are some of the arrangement that has been put in place but as far as our objectives is concern we have achieved it. No ,we do not have any objectives as far as incidence of these diseases is concern.

N:So which of these interventions that is the education, and mass drug treatment where initiated by the NTD program?

R:Long before the NTD program started these programs where in place.So the NTD program is lending a support because the nature of the support involves a lot of funds and with this dwindling funds situations we need external help.

N:So with this program do you have timelines with respect to when to stop?

R:No,infact we do not intend to stop because diseases control is a spectrum of intervention and we have just taken a band of that spectrum,so if we stop ,we wouldnt be achieving anything because the drug distribution goes alongside with other interventions and so long as this other intervention is not going on then the drug treatment would go on for a long time.

N:So are there any other intervention or activities beyond the one you have mention that the district have initiated as part of the control of these diseases?

R:No,I don’t think we have initiated any intervention beyond what we have discussed.

N:So what is the role of the district head administration in the control of phyliariases, chitosomiasis,STH and soil transmitted illment and onchosiachasis like you have mention?

R:First of all ours is to co-ordinate the activity, it is also to organize and also to train the human resource to make sure that the distribution of a treatment and the distribution of the drug goes according to schedule that have we don’t have unnecessary shortage in terms of distribution and things like that, and ours is also to ensure that the target group, the communities embrace it, the communities sees it as something to improve their health condition. So we also ensure that other decentralize agencies also learned support. So we bring other agencies onboard, those who can play a role in advocacy, those who can put their resources at our disposal to ensure that we achieve the objective.

N: So what is the role of the NTD program in the control of these NTDs in this district?

R:The role of the NTD program first of all is to bring in additional resources, funds. To bring in additional resources, so that is essentially what they do but as you know, the resources will be used for various activities, so what they do is essentially bring in additional resources for the program.

N: Are there any, or let’s say which agency or organization do you consider ultimately responsible for the control of this NTDs?

R: Well I consider the Ghana health service as ultimately responsible, yes the Ghana health services.

N: And when you look at the NTDs in your district, would you consider may be the district ultimately responsible or lets say the NTD control program or the region, what will you say?

R: The district is ultimately responsible for the control of NTD program in the district, the district health administration is ultimately responsible for the program in the district, in fact we are responsible we are accountable to you know, but we need results you know the nature of the health delivery system is such that resources have to come from beyond us, so that we will be empowered to put into practice what we have put on paper in term on plans and things like that, yes.

N: And looking at still responsibility, which officer or officers would you consider ultimately responsible for the control of NTDs in this district?

R: The district director of health services, he is ultimately responsible. The district director, he is ultimately responsible. Of course he works with a team supported by the disease control officers, the field technicians, the records guys and other colleagues. doctors, nurses and the rest of them.

N: So what are the reporting channels for NTDS from the community to lets say the regional level,in the district when it comes to NTDs reporting am sure it happens in the community, what are the channel that exist from the community up to the regional level.

R: We have volunteers in the community and the volunteers have a supervisor, so a number of communities will have one supervisor and that supervisor will collect the reports and send it to a coordinator who is normally at the sub district and he will in turn transmit it to the disease control center who will compile everything and then submit it to the regional level.

N: So this are the channels but specifically who are this supervisors, what category of stuff are they at all this levels of transmission of data, who are the specific officers?

R: They, some of them, they vary. They are community health officers, they are physician assistance as we call medical assistance…

N: At what level?

R: At the sub district level, at the sub district level. So we have the community health nurses and physician assistance acting as coordinators and supervisors at the sub district level and then you have the disease control officer who is at the district level, but between the interface of the supervisors and the disease control there are coordinators, there are layers of coordinators, they are officers at the district health administration, for instance the health information officer, the public health nurse, the nutrition officer, sometimes we even use the hospital staff, so they coordinates all for us and they channel it to the disease control officer. So you have volunteers and then volunteers send their reports, they capture data send their reports to supervisors who are community health nurses or sometimes physician assistance who are in charge of the health centers and then they also give theirs to coordinators who are also health information officers, nutrition officers, public health nurses or hospital staff and then they eventually gives it to the disease control officer.

N: Who also submit it to the region.

R: The region yea.

N: So what is the role specifically of district directors in areas like yours where NTD is endemic, meaning in the control of this NTDs in the district so what role will the district director be?

R: Well district directors role first of all is to ensure that in terms of drugs we’ve had the requisite number of drugs in terms of sensitizing the communities creating the awareness of an impending exercise he ensures that that is done, he ensures that generally the work goes on well because he designs the plan with a team, everybody makes an input and he ensures that what is put down is implemented to fully to achieve the intended result. So these are the roles of the district director relative to NTD.

N: Now for the NTD program, what role do you think they play in achieving the control objectives in the district, the NTD program, they look like a national program but this is something they are suppose to be doing. What role do they play in the district in ensuring that the treatment objective and the thing that are achieved.

R: They play a supportive role, normally when the NTD program officers come, they hush or reemphasis on the objectives and so in the course of the interaction they impact to the officers the level of seriousness and the need to realize the intended objective, so they are if you like they supports, they influence, they inspire, they motivate the workers to do the work in a manner that is required and they also funds to support the whole exercise.

N: Ok talking about fund , we moving to financing now. So what generally are the sources of funding for NTD control activities in the district?

R: First of all one, it comes from the NTD program it self, two we solicit funds from the district assembly, the district assembly lends support to it, and three the hospitals also supports us. So we get it from the NTD, the district assembly and from the hospitals also support us.

N: what about general Ghana health service pool funds, are they anything that comes from that to support NTD activities?

R: No, Ghana health service funds are year mark funds.They are rely fix ,they are use for intended purposes.So that is why we actually go for support from the hospitals through their internally generated funds,

N:So are there situations where funds from NTD’s control activities could be use to support other priority interventions in the district?

R: Yes,infact we do that we couple other activities to the program because we know that we are resource straped so we do the coupling with other activities.For instance tuberculosis and this defaulter tracing and rest of them,we have been able to reduce this defaulter tracing but when they do occur we use these as means or vehicles to address them.So you are going to aparticular area,you have been given a vehicle it has been fueled, you take advantage of your presence there not only to sort NTD problems up but to also look at other related health activities there.

N:So does it apply the other way round,lets say funds for reproductive activities,TB,AIDS,Malaria are also use to support NTD activities.

R:Yes,normally sometimes we even pre-finance.there are delays in the release of funds so we

have pre-finance so we have to source for funds from other programs so we do so knowing that there is a pledge to pay back.So it is not relly using other funds to do it but we use others to do it knowing that what ever cost we incur, would be defrained by NTD program.

N:But is there any situation whereby some of the education or something is funded by other sources rather than just NTD year mark funds?

R:No,as I said the assembly helps ,the hospital too helps.Normally,we don’t use other funds to do those problems

N; So we move to the next section on planning. So what is the role of the district health administration in accessing/accessment of the resource that are needed to conduct NTD activities in the district?

R:In planning we are able to a large extend, cage the resources that we would require for the whole activity so the district health administration give an estimation what we would require to carry out an activity.Often as always what you will require is not what you would get because what you would get is determine what whoever is given you.The district health administration accesses the resources that is how sometimes we are able to determine or detect short forms to get additional funds from the hospital and the assembly.

N:So does the regional health administration also do some accessment of resources they think the district would require run those NTD activities?

R;Yes,region also helps, region normally helps with vehicles for transportation,you know our area has a very difficult terrain,Infact some of them you have to access them trough Tarkwa by going through Bogoso and Prestia so it a very difficult place to access,so under such situation the region help us because they have an appreciation of the difficulty relative to transportation.Yes they also make estimate the needswith respect to what NTD’s are in the district.

N:What about the NTD program,do they also make an accessment the resource that you need for the activities?

R:No,for the NTD what they do is that they bring their funds and the funds they bring also do not even consider number the volunteers.Iam saying so because the unit that is been given to a volunteer is determine by how many volunteers you would need.So sometimes they may decide ,for instance you say you need ten and they are saying you should use six.

N:So when it comes to issues of priority setting with regards to NTD programs ,what role does the district health administration plays in your district. Priorities in terms of when to treat ,which population to treat?

R:No, in terms of when to treat and the population to treat,how far you should go with the treatment and perhaps duration,No we don’t do that,we do exactly what NTD program suggest.So the priorities are set by the NTD program.And as for the regional level I don’t whether NTD program set those priorities too.

N:What is the role of the district health administration in the allocation of resources for the NTD control activities in the district

R:When it comes to disbursement,appropriation and distributing it to the various grouping.In terms of grouping,the district head administration see to it that every group,every sub-district get it fair share of resources to implement the activities that we have set out

N:What is the role of the regional health administration in the allocation of resources for the NTD control activities in the district

R: Normally they give us a spreadsheet but as you know the spreadsheet is a guide and you are not compelled to stick to it because perhaps you are familiar what happens on the ground and you can vary it but it is a guide and in terms of allocating resources they give that guide.Often,the allocation is done using a number of critirial,one of them is population so in terms of population and distribution in various sub-district ,they also have our population so they know.So they may be using population but in terms of terrain and accessibility then we know these people need more here because eventhough the population is not that big there is difficulty in accessing

N:The NTD program does It also play any role in the resource allocation for NTD activities in the district?

R:Yes,they allocate the resources base on the facts and figures that we have given them base on number of sub-district, base on number of volunteers and base on the number of target group.so that is what they do.And also the duration, and also have a role to play in the resource how much to give.

N:So the next section,we would look at service delivery,delivering the mass drug administration and health education. So what category of staff at the district health administration work on NTD control activities?

R:Everbody,the health information officer does that ,the disease control officer ,the nutrition officer,the field technician , the accountant,the district.Everybody ,has a role to play and these are all people employed by the Ghana health Service.They are not dedicate to NTD activities but rather they are muti purpose.

N:Are any of the NTD control programs undertaken together with other disease control activities in the district?

R:Yes,like TB

N:What referal options are available for NTD cases that are identified in the peripheral health facilities?

R:There is a referral system for diseases and normally depending on how the disease come to our notice through community health nurses or community health officers at chip centers interacting with the people then they go through the normal routine.So for example like somebody is having hematurial and quickly they would refer,they may know that this is chistosomiasis but invariablely they do refer and when they refer it comes to the hospital and the hospital go ahead and treat it

N:So what are the essential logistics and drugs required for NTD control activities in thedistrict?

R:First,we need vehicles and we also need the drugs when it comes to alvemetin for oncho and plasofantel for chitosomiasis. And apart from that it also involve training people, sometimes is a refresher training depending on the nature of volunteer, sometimes there is an attrition people leave,new people comes so you have to train and retrain and then we also have to synsithesize.There are various media available to us,in a typical village they call it the gong beater and others a PAsystem that they have we use that.And we also use the Fm station because the listernship for the Fm cut across districts, sometimes ajoining districts help each other so you take advantage of this.Some may say they heard it from Ahanta West or Elembele East etc. so this is how logistics are organized.

N:Does the district heath administration play any role in the procurement of this drugs you require for NTD’s?

R:No,we do not play a role we essential receive and then distribute,They also do not determine how much drugs they need to buy

N:What role does the district health administration plays in the storage,distribution and delivery of these NTD drugs to the target population.

R;Yes,we receive the drugs when it comes from the region to the district and base on the distribution plan we distribute it to the sub-district and that in turn gives it out to the various volunteers that they can use,they give them on daily basis so that we don’t choke system so this is the trend of distribution and so normally there is an amount at the district and depending on storages we respond appropriately.We go for the drugs at the region and viceversa and when we get it we store it at the district stocks.

N:So we will move to the next section which is monitoring and evaluation.So what monitoring and evaluation activities are conducted by the district heath administration to access the achievement interventions objectives as far as the NTD’s are concern?

R:First of all the supervisors are suppose to monitor compliance,compliance to doseage,are you giving the appropriate doseage and there is a format for example sample 20 for every volunteer and you do the same for another one and make sure the volunteer is doing the right thing and also in terms of tallying/recording they are doing the right thing.So these are the basic things that are done on the ground and they transcribe it in the forms which would eventually reach the control officer.infact we don’t purposefully do that but there is always a team that comes to do a survey they normally com from Kintampo.So they do a survey to find out the coverage and see whether it matches what we have put together as administrative coverage.

N:So do you the NTD program itself conducting surveys or laboratory testing to find out how effective the treatment has been?

R:Yes,they come the NTD officers come because for Onchosachiasis I think they come for skin survey and tests.When they come they inform us and the district is involved because they may not know where to go ,they may know the community on paper but they don’t know they also make sure the people they are coming to see are there when they come so the district organizes these things so that the purpose for which they came is achieved

N:What ICT infrastructure,here I mean computers,internet access,websites ,telephone is available at the district health administration to process and store and communicate all NTD related issues and data.

R;Now,I think the ICT has penetrated the institution and individuals have take advantage of it to have access to internet and all manner of applications that they use to analysis data and submit their report in a timely manner.So people have access to computers and all the rest.The ICT infrastructure is been shared for other programs as well and some of them we receive them from the region and some of them the district bought it for the offices

N:What is the role of the district health administration in data collection for NTD and MNE activities in the district?

R:Yes,the district does that for instance if we do drug distribution we in terms of coverage to find out whether we have achieved set target,it is the responsibility of the district and the district does that but when the NTD officers come in,they do their own thing and compare with the administrative figures that we have.So they are using different methodology to come up with coverage and we also use a different one.So since we do coverage the data we analysis it.

N:What role does the district health administration plays in the monitoring and evaluation activities of NTD control activities in the district

R:Yes,the region has a supervisory role and they also ensure that we have data quality .The data quality that is when we submit they give us feedback in terms of figures that might not be matching.So they look at the data very well and gives us feedback on what is not appropriate.

**Demand Generation**

N: We are on the last section,the demand generation,so what activities does the district health administration undertake to improve coverage and increase demand for NTD interventions activities in the district?

R: The district does a lot in terms creating awareness, sensitizing people. When we are going to have a program like that we keep the community informed beforehand, maybe two weeks before hand so that they would be ready to receive volunteers when they come. So that is essentially what the district does and as I alluded to earlier, we use our community health nurses who come into direct contact with them. We use the existing communication system in the community so they beat gongon they use their own PA system and we also use the fm to get them mobilize.

N: So what is the nature of financial incentives available to staff of the district health assembly to improve coverage of NTD interventions?

R: The part we can do is to ensure that the district assembly and the hospitals are giving us more resources, because as for the NTD theirs is a given so perhaps we can’t do much but for the district assemblies and the district hospitals they can raise their level of support the funds that they use to support us with. So once they do that we can also do more to increase the coverage.

N: So are there some financial incentives for the staffs to encourage them to work so that coverage is increased

R: The existing reward system is the regular reward system, there is an allowance that is given to an officer depending on the nature of his work and that is regular. So that is what is done that is what is done, we don’t do more than what I said.

N: And what is the source of these resources that is used to motive the staffs?

R: As I said earlier, they come from the NTD, they come from the district assembly, and they also come from the hospitals as IGF to support us.

N: and then when it comes to, am aware that some non district health administrative staff take part in this activities, what form of financial incentives do they also get?

R: They get the same as the regular health staff gets so long as those whose nature of work are the samething they get the same level of allowance regardless of where you are coming from whether you are a Ghana health service staff or non Ghana health service staff so long as you are a supervisor you get what the supervisor get, so long as you are a coordinator you get what the coordinator gets so long as you are a driver you get what a driver get, and it has a margin this is a kind of intervention that require mobilizing resources beyond what the district health administration can master so we tap into other decentralize agents to give us vehicles to put their drivers at our side, so we ought to treat them well so that they can embrace subsequent programs.

N: Could you mention some of the non NTD staffs that you use?

R: Agric staffs we use the Ghana health education staffs we use non formal community staffs these are the, we even use the district the national health insurance staffs to do the work.

N: and for this incentives that you give them also, from what sources do they come from?

R: The same source.

N: So when it comes to this sensitization activities and health education which staff at the district health administration does that, yes conduct that?

R: The health information officer does that, we have very experience field technicians they also do that and the disease control officers. Sometimes we enlist support from the assembly, there is this group and decentralized agencies that work with the communities regularly so they know the community nuances. When we explain to them exactly what we want/objective of what we want. They go with our officers and give the message.

N: Do these staff you have mention like disease control officer, information officer, field technicians develop materials, conducting health educations and social mobilization activities in the district

R: Yes they do. Actually the district has a social mobilization and community sensitization materials, it is standardized. So they all have the exact message to give out to the communities. The posters, flyers are develop above us we receive from region and I would have taught the region also receive from national level.

N: Is there a health promotion unit in the district health administration and what role do they play in conducting social mobilization activities.

R: There is no specific health promotion unit but there are various category of health officers who are involve in health promotion .You know because of the nature of health system at the district level, various category of people play roles beyond what is normally required of them .So a nutrition officer would not be concentrated on only nutrition he would be involved in prevention, health promotion and the rest of them. So they are a number of health officers at the district , who are involved in health promotion activities.

**Section H: We are rounding up**

N: How long have you been a district director in this district?

R: Seven years.

N: Before then what was your previous appointment?

R: The same district director I was at Enchi

N: Thank you very much for this interview and I am very grateful for your time. Do you have a comment or questions before?

R: No, I am very happy. Very happy in the sense that you have explored various aspects of the work and as challenged me to look into those areas very well and also share with my colleagues. The officers I work with about the areas that we have dealt with and how we can improve the nature of work relative to those areas. So it has been a very useful encounter and I am also happy

N: I also thank you.

**Interview Six**

N: Good morning once again

R: Good morning

N: I am a student of the university of Ghana school of public health, I’m undertaking a fellowship there and I’m conducting this study as part of the course requirement and once again I want to thank you very much for your time and indeed your input as the **District Disease Control Officer** is very important for this study. Your responses can be as brief as possible but please elaborate where you think it is necessary to do so. Once again I am Ernest Mensah and the interview is expected to last at most an hour. It will be recorded and then transcribed later for analysis. Please do you have any comments before we begin?

So to do this study the interview has been divided into sections. I am looking at the NTD situation in the district. I am looking at the issue has to do with governance or how you manage the disease issues and I am looking at the issues to do with financing , the planning, how you plan your activities and then service delivery, how you deliver the services and then monitoring and evaluation and then also social mobilization . So the first section is on neglected tropical disease situation in the district.

N: Are NTD’s among the priority diseases in the district?

R: Yes, I must say they are priority diseases in the district because some of them are very eminent in the district so its our priority.

N: So which of them are found in this district?

N: Elephantiasis which is lymphatic filariasis that is the most important one you can see around in this district. And few ones like schistosomiasis is around.

N: Do you also find soil transmitted helminths around?

R: Yes we found them too.

N: So which of the are considered endermic in the district?

R: The elephantiasis

N: So generally what interventions are in the district to control the elephantiasis you have talked about

R: As for the intervention one I know of is from the national level, that has also been part of the district. Such as every year we distribute this albendazole and ivermectin to the whole population so that we prevent new infection and also avoid other from getting the disease too. So that is what we are doing every year

N: Which NTD control intervention in this district where initiated by the NTD control program?

R: The first one I talked about on the elephantiasis and then soil helminthiasis and schistosomiasis that is the one that was initiated but as for this place we don’t have trachoma here even though the other drug is combination for both elephantiasis and trachoma I think it have a dual purpose. Since I came here too there was these NGO World Vision Ghana, they were also supporting but I can’t tell whether they initiated it or the Government who brought it and they supported it.

N: What are the general objectives or expected outcome of mass drug administration that is conducted here

R: It is expected that some time to come or we should say it now we won’t see any infection if a lot of people is patronize it we won’t have any infection but rather we would be managing the old infections. That is the main objective of the mass drug administration.

N: Are there any time lines for the achievement of the objectives .

R: Timelines for me I see it that in the next ten I think we should eliminate this disease from the district and as I said they should see the old infection and not the new ones. I think these program has been here for quite some time so we would leave it to the expects who can come and do surveys to determine whether the infections are still around.

N: So these about ten years you are talking about is what the district has decided or the region or NTD program who have decided?

R: This is district specific.

N: Are there any other interventions or activities that the district or region is undertaking outside the NTD mass drug administration.

R: No not rely we all depend upon the national activities.

**Section B: Stewardship and governance**

N: So what is the role of the district disease control officer in the control of LF schisto that is STH in the district?

R: The role of the disease control officer is to ensure or see to it that we are part of management system that can help to reduce the burden of the disease in the district and also prevent other from getting infected as well as and also do a lot of education on the mode of transmission, how it is acquired and when you acquire it too what you are supposed to do. So mainly on education and managing the diseases would also play a lot of role.

N: What role do you play when it comes to mass drug administration as you being a disease control officer?

R: When it comes to mass drug administration the main role is to assist in training the volunteers as to what they would do on the field and also going round for proper supervision ensure that what is being taught is done on the field and also find out whether others are reporting of side reaction and what we can do for them.

N: What is the role of the NTD program from the national level in the control of LF and STH in this district?

R: Their role in the control is to make sure that the drugs should always available and especially timely. We know that every year or based on timelines we are supposed to do the administration so the drugs should be there so that we follow the time schedules that is their role and also helping with funds so that the program can go on successfully.

N: So which organization or agency or program do you consider ultimately responsible to make sure the LF and STH is controlled in this district?

R: I think is the district health directorate is the main agency or control because we are the first line of contact when these diseases are around.

N: So in the district which officer/s would you say they are ultimately responsible for the control of LF and STH in the district?

R: Those responsible are the district director and disease control officer but as a district we always work as a team and each has its role they play. My major responsibility would be to make sure that these diseases would not be of public health importance for some years to come.

N: What are the reporting channels for LF and STH epidemiological status and control activities in the district, looking at from the affected communities to the regional level what are the reporting channels?

R: Ok, we have community base (CBS) volunteers in the district, every community has it and they have been trained to identify some of these diseases, so that anytime they see this diseases in the community. They are supposed to report to their immediate CHO’s supervisor and then she in turn inform the sub-district head and it would come to this place. And aside from that, we have forms of diseases that we have these diseases captured on it, so each month during our reporting if we don’t have then it is zero, or if we have we take note. Every month we submit report, even though it is not NTD specific report we have a report for the district, different ones that captures NTD’s in it.

N: So which of the officers are responsible for doing all the reporting at all these level?

R: Ok, at all this levels we start with the CVS volunteers ,and then when you to the sub-district we have field technicians in every sub-district and they would take it from there. Sometimes if it goes through the OPD then the medicals record assistants they would pick it in the morbidity and we would later have it for the month. Then, when it comes to the district I am responsible to submit it at the regional level.

N: What about after you have done an MDA how does the reporting go and who are responsible for all the activities?

R: For the MDA we have community registers those ones are used by the volunteers and the next level, that is the supervisors for the various community they have an MDA form and at the end of the program they submit the books to the sub-district for compilation and put the summary on it before bringing it to the district and I would also do my summary before I submit to the regional NTD coordinator.

N: What is the role if any, of the district director and district disease control officer of LF and STH endemic areas in ensuring achievement of objectives?

R; I think that the as district director he has a major role to play because he is the head of the district. He and I plays side by side, he has to ensure I do my work properly so that everything moves successfully.

N: What is the role of the NTD program in ensuring that the objectives for LF and STH program are achieved?

R: The role they play is that ensuring drugs are available in the country or district anytime we are having an MDA and also funds are available.

**Section B: Financing**

N: So we would look at financing issues. Which generally are source of funding for NTD control activities in the district?

R: The NTD program for the national level they are the main source of funding and sometimes we get help from somewhere like the World Vision Ghana who have just moved out three months ago.

N: How is the NTD program funded with respect to the general GHS pooled funds and the district earmarked funds?

R: In fact I don’t know much about that. All I know is that the money comes from the NTD.

N: So are they situation where funds for NTD control program are used to support other priority interventions in the district/

R: No, there hasn’t been any of this situation that I know but as for me what I know is that funds brought for this program are use for that program.

N: Are there situation where NTD programs have been supported with general district health administration funds from other priority diseases like Reproductive health, TB, AIDS, Malaria?

R: Sometimes, it depends on the availability of funds and the funds we get from the national level if its not enough and we can do something about it. for instance like the last NTD program the money was very small and the volunteers were complaining so we gave the incentives aside from what we gave them from at the national level. But when we it comes to the running cost like the stationery and other things is the district that funds it especially with NTD’s but with other programs it is not like that.

**Section D: Planning**

N: We go to the next section on planning. So what is the role of the diseases control officer in accessing the resources that are needed to control LF and STH in the district?

R: I should make sure things we need to work with are enough and if not how we can manage it. I see to it that as for these particular community due to their number they would need this number of volunteer or that and also the logistics which we are going to use are all a available so that there would not be shortage anywhere so infact I take part in the planning of logistics and whatever would be needed

N: Does the regional health administration also take part in determining how much this district or community needs to run the program?

R: Yes they take the bigger part because they do the funds distribution and allocation of logistics based on your district population and we it comes down we also break down it to suit every community.

N: So when it comes to accessing the resources needed to control NTD, what role does the NTD program play?

R: Their role is to ensure that the resources are available and in the accessing the NTD is a nation program so I think they also some accessing in the region.

N: What role do you play when it comes to NTD programs in setting priority in terms of what to do?

R: Yes, I play a role there, before the program starts we have a meeting and plan and also give roles to my supervisors play so based on that I think we achieve good results.

N: Does the regional health administration plays a role in setting priority in terms of when to do what and also determine the process?

R: They do because we always go in line with them and also there is a timeline for conducting programs so during regional meetings we are given a timeline so base on that we are supposed to work within that time.

N: Does the NTD program also play a role in determine and setting priority in how the work needs to be done?

R ;Yes ,I think everything comes from them before the region gets to know of it and even during planning at the regional level they always there to plan with us.

N: What role do you play in the allocation of resource for the control of LF and STH in the district/

R: The role I play is to make sure that the resources are allocated very well to prevent any shortage. Whenever we get spreadsheet for funds and logistics, I make sure that base on sub-districts and communities, I allocate to each community according to the population and number of volunteers.

N: What role does the regional health administration plays in the allocation of resources?

R: They also play a role they should see to it that all regions has logistics and resources so they also sit down to do the allocation

N: What role does the NTD program play when it comes to resource allocation?

R:They also play almost the same role because they do the allocation at the top, then it comes with the spreadsheet to the regional level and on the spreadsheet eventhough the region would not go specific it is within their spreadsheet.

**Section E: Service Delivery**

N: So now we are looking at service delivery. What category of staff at the district health administration work on NTD control activities.

R: All the staff, I said here my district director likes us to play as a team so even if there I would be a program that is for diseases control and even you may be the head, all other unit heads would be in support of the program till we finish, He also goes round when each officer is having a program and support.

N: So for all of you who take part in the program which organization employs you?

R: The Ghana Health Service

N: Do staff working on NTD programs share offices and office equipment, vehicle ,medical stores with other staff of the regional health administration?

R: No, we don’t share offices but sometimes when the need arises and we have to be in a group to do discussions then we should all be in one room but we all have separate computers and offices.

N: Are any of the NTD control activities undertaken together with other diseases activities in the district?

R: Yes when it comes to education when we have to do community sensitization we merge with other diseases which are of the same origin

N: What referral options are available that are identified in the peripheral facilities in the district?

R: Within the district they have been made aware that treatment for NTD’s are free so sometimes they don’t have to go through bureaucratic system. So sometimes they come to the health facility and report themselves and there are times that our own health workers accompany them to the health facility to get treatment. The district hospitals also do a lot referrals when they cannot tackle some cases.

N: So what essential logistics and drugs are required for NTD control activities in the district?

R: Drugs like Albendazole, ivermectin and praziquantel is also needed since see one or two schistosomiasis around. Some other logistics sometimes, even though is not provided but we provided are the management of elephantiasis we need soap, clean towels and others.

N: So what role does the DHA play in the procurement of these drugs and logistics that we use.

N: **As for the procurement we do not play much role, the only thing we do is to use our vehicle to go for our part whenever these drugs comes to our medical stores.**

N: What is the role of the DHA in the storage, transportation and distribution of these drugs and logistics to the district?

R: First, we have to go for the logistics and drugs from the regional medical stores with our own vehicles and when we come ,our own vehicles will convey all this drugs to the sub-district. And after the sub-district has done the distribution, is with our own vehicles that take it to the volunteers of the various communities.

**Section F: M&E**

N: So we would now look at monitoring and evaluation. What monitoring and evaluation activities are conducted by the district health administration to assess the achievement of NTD objectives in the district?

R:The work is mostly done by our frontline workers like our CHO’s and field technicians ,they are supposed to find out if people are still reporting of these kind of diseases so they pick information from community base surveillance volunteers. For some time now we have not any seen new cases so I think that everything is on course.

N: Like you are given out drugs for example do you do anything to find out whether people are actually taking the drugs?

R: The drugs is taken on the spot that is direct observe therapy. That is how we do the distribution so people do take the drugs except that sometimes because of side reactions people refuse .Some of them we can’t do anything about it. Some of them when you talk to them they do take it but majority of the population are taking the drug base on the documentary we have being shown them and what they have seen.

N: So what ICT infrastructure are here I mean computers, internet access, websites and telephone which are available at the district health administration in processing and storing and communicating NTD related reports.

R: The main thing is that we have the hard copies and store the soft copies in the laptops. Yes, for internet access we use modem but we don’t have a website and we also have telephones too.

N: What is the role of the district health administration in M&E activities of NTDs in the district?

R: Yes the district plays a role because in this district we always work as a team. These NTDs especially the elephantiasis is a priority diseases for the district so each officer is aware because is a priority whenever they go out during their monitoring ,even if it is not part of their schedules they take time and do some small monitoring on this diseases.

N: What is the role of the NTD control program in M&E activities in the district?

R:The NTD program also plays a role because whenever we have MDA even though they are supposed to be in Accra they and then visit each district to find out what is happening if possible they even visit some of the communities to rely do some small monitoring and evaluation before they leave.

N: Have they do surveys or blood samples to check whether the drug is effective or not.

R: The NTD programs have conducted these surveys but we are yet to get the results. Other people from school of public health has also conducted some and many organizations .The district also get involved because before they conduct the survey the organization seek our concern, so when they come we are aware and join them when the time is convenient. As for the region, I don’t know whether a survey has been conducted before since I have been here for three years now but they do M&E.

**Section G: Demand Generation**

N: So now we are looking at demand generation or social mobilization. What activities does the district undertake to improve coverage and increase the demand for NTD interventions in the district?

R:When it comes increase demand, everything comes with a spreadsheet sometimes you relies that the number of volunteers they give us is even smaller than the number of communities we have so if we have funds or sort the district assembly to help then we increase the number of volunteers so that we have a lot of people being covered, which would increase our coverage ,aside from that we do a lot of public education before the drugs is being taken so base on that people are aware and they take the drug too.

N: What mediums for example do you use for the public education?

R: As for these community we don’t have fm station in this district but I think almost about 80% of other communities have this community information, the local ones that is the main information we use to do the education and we also use the vehicle with PA systems to go round the communities.

N: What is the nature of financial incentives available to staff of the district health administration to make sure that they improve coverage for NTD activities?

R: Yes, some small funds.

N: What are the sources of the financial incentives?

R: The sources is mainly from the NTD program

N: What is the nature of financial incentives for non-staff of the district health administration but participate in the NTD activities?

**R: Yes, the volunteers the NTDP takes care of their transportation after they have taken part in the activities. And the source of financial incentives is from the NTD funds**

N: So which staff are involve in developing material and conducting health education and social mobilization activities for NTDs?

R: We have a social mobilization team in place and the same team works for all other programs. Whenever we are supposed to do the MDA, these people take up and it’s made of the public health nurse, the health information officer and one technician from each sub-district.

N: Do you have a health promotion unit?

R: Yes, we have. They do take part in the mobilization activities but one issue is that the head of the health promotion unit is not fluent with the Twi so sometimes when we go to the community he cannot express himself very well, so he gives as a lot of ideas and we implement it for him.

N: DO you have materials that you use to educate people when it comes to LF and STH?

R: Yes we have posters and we have a lot of books on LF especially that we use and also documentaries and films that we show to them through the PPAG

N: Do you develop these posters and documentary at the district level

R: As for the documentary that is available, I had it from the regional level. I think the national came to do a program here. As for the posters and everything we get it at the regional level stores.

**Section H: We are rounding up**

N: How long have you being a disease control officer in this district’

R: Three years

N: And before that what was your previous appointment?

R: That is my first appointment.

N: I would like to thank you for participating and the information you have given me is very valuable. Do you have any comments?

R: No, I don’t have any comments

N: So thank you very much.

R: You are welcome.

**Interview Seven**

N: Good morning again I am Ernest Mensah, and I am undertaking a fellowship at the University of Ghana School of Public Health and I am conducting this study as part of requirement for the course, and want to thank you very much for accepting to be interviewed. Your inputs are very important as the district director of the Ahanta west district. Your responses can be as brief as possible but please elaborate where you consider appropriate to do so. The interview is expected to last about an hour and it will be recorded and transcribed later for analysis. Please do you have any comment or questions before we begin?

R: No

N: Thank you very much. To look at this study, I’ve divided to it into the NTD situation in the district. I will also look at the stewardship issue that has to do with management of the disease; I will look at the planning, the financing, monitoring evaluation issues and also demand generation that has to do with social mobilization. So the questions will come from that angle, so first one am just looking at…

**Section A: NTD Situation in the district**

The first section is on neglected tropical diseases situation in the district, please are NTDs among the priority diseases in the district?

R: In this district I can say yes because we have lymphatic filariasis, which is very prevalent here, we are not getting new incidents case, throughout the mass drug administration. Some few years back buruli ulcer was also a problem, but now we are not seeing new cases but occasionally some of the old cases are managed directly from Accra. Dr Ampadu and his people come and once they are identified wounds we do the swap.

N: So which NTDs are reported in the district?

R: Mainly is buruli ulcer and lymphatic filariasis.

N: And which of this is considered endemic in the district?

R: lymphatic filariasis.

N: And will u say the schistosomiasis and STH are also reported here?

R: Schistosomiasis used to be a problem and Ewusejo and Abra but because of persisting education and for prevent to prevent their children from swimming in the river, you don’t see much now. Helminthiasis I will not be able to confidently say yes or no because in the CHPS compounds and hospitals you see a diagnosis of helminthiasis but in the CHPS compounds we don’t have laboratories to test, in the hospitals too some of the helminthiasis that they diagnose when you follow up you see that this symptomatic treatment, so I don’t know if its real helminthiasis or not.

N: So generally what interventions are there in place in the district to control the NTDs that you have mentioned?

R: with the lymphatic filariasis there is mass drug administration that is that is made up of ivermectin and albendazole which is done yearly then during school programs too we do mass de-worming, child health promotion and some school programs, if we have some drugs remaining we do mass drug administration for the school children. Yes.

N: So which of these interventions that you have mentioned were initiated by the neglected tropical diseases program?

R: I don’t know but I guess the mass drug administration of drugs of ivermectin and albendazole has a direct link with national NTDP headed by one doctor Biritwum.

N: Yea that’s the NTD PM.

R: So I guess the money comes from W.H.O and its outfit in Ghana health services to support. Because they are funds from national depot.

N: Ok, so they actually initiated the mass drug administration that the district is running.

R: It is not a question of they actually initiate but it has become a routine that almost every year for the past ten years, we’ve been doing the mass drug administration.

N: So what are the general objectives or expected outcomes of this mass drug administration in the district?

R: Is to reduce the incidents of NTDs that I have mention and in some cases also reduce some complications that might be associated with blocking of lymphatic vessels in the children also to prevent anaemia and other nutrition related problems.

N: Are there any time line to achieve this objectives in the district? Are there any time line to say we want to achieve this at this point at this time or anything like that?

R: Yes, because we are all talking about millennium development goals 2015, if somebody has well developed a, elephantiasis definitely it will affect the persons productivity and the person will not be able to earn some money so it will automatically go to perpetuate poverty, you know if you see some of the few scrotal swellings that I have seen then men are really pathetic, but that is 2015, after 2015 whether all those will be coming in to support the program is another question altogether, **You know the biggest problem is that there is too much verticalization of the health system, there is the need for integration that can take care of all aspect of health care not looking at malaria from the top you know TB,HIV, You know.**

N: so for the district, the main one it’s let’s say the millennium development go but they haven’t set up stuff like lets say in the nest five years or something like that .

R: No but I have not, the reason why I am saying this, is that I don’t know how long the mass drug administration is going to continue.

N: Okay, Okay

R: **And if you take things like helminthiasis , you know you the warm after some times the will be re-infected and you the you worm and they will be re-infected and it cost .I don’t have the resources to provide and it the program ends and we don’t finds a way of integrating in to current health system .I don’t know it will be difficult to say.**

N: Are there any other intervention activities initiated in the district or in the region to manage the NTDs that you have mentioned outside the ones that NTD control program leads.

R: Outside the NTD program where we go we talk to them about washing of feet You know especially to those who have already been infected you know how to dress their wounds so that there cannot be complications and we has so video clips of how some or few of them have been amputated because of complication of wounds and this is to prevent them from getting into serious complication

N: Thank you very much.

R: And sometimes too, because of the insurance we advice them to come for hydrocoelectomy.

N: I think for this particular study, I think we will limit the discussion to the LF and then the STH’s soil transmitted Health that you found here. The question I asked previous I just wanted to probe a bit further apart from the mass drug administration that the NTD program brings ivermectin and albendazole and sometimes you use some for the deworming, are there any other intervention or organizations that are here and trying to address this saying

**R: Organizations know, but when the Ghana Health Service try to do, you know elephantiasis is a transmitted by a form of mosquito so when we are doing our education on the use of bed nets we don’t want to tell them the bed net prevents malaria but also tell them that it also prevent them from getting elephantiasis by killing the mosquitoes that bit them to introduce the bacteria into them.**

**Section B: Stewardship and governance**

N: Thank you very much. So then we move to the next section that deals with stewardship and governance so what is the role of the district health administration in the control of the elephantiasis and STH in this district?

R: The role of district health administration is basically that of co-ordination, they bring the things from the national level and we coordinate and make sure that the drugs are distributed from the DHA’s to volunteers who distribute them and then at sub district level the sub district heads will be monitoring for ADR/adverse drug reactions and ensure that the people are taking the drugs.

N: So what is the role of the NTD program. In the control of LF and NTDs in this district

R: The role , not a very simple question.

N: The national program what role do they play as for as

R: So far as I am concerned , once a while if there is a research that should come here or the KCCR people, come here from Kumasi and tell , but most of the things that concern the mass drug administration we are invited to Takoradi and say this program is going to come on this time and you should get ready to distribute the drugs, I don’t know whether is the national or who or what.

N: That’s the national program conducting those thing ok

N: So which agency organization or program do you consider ultimately responsible for LF, STH control in the district?

R: That responsibility lies with Ghana Health since its part of the this thing

N: so Dr. Sutherland with respect to the responsibility of the control of LF and STH in this district

R: The sole responsibility lies with Ghana Health service and they delegate us to do because of the decentralized periods.

N: Right, Right, would you say that so it is from the Ghana Health Service. You are taking lets say the NTD program as representing the Ghana Health Service or you think it is the responsibility of the district for example to make sure that it is controlled in the district.

R: You see, if I even say Ghana Health Service even wrong, because it’s the ministry of health that develop the policies and we are suppose, we are service providers and we implement it , so so far as we concern. Since it’s a disease, we are supposed to control it

N: Alright, thank you , and then which office or officers do you consider ultimately responsible for the control of LF and STH in the district?

R: Which officer?

N: Yea or officers do you consider ultimately responsible

R: It’s the responsibility of every health worker if they identify them to ensure that are treated.

N: Alright,

R: Including volunteers that we treat.

N: Oh okay, but you don’t think that maybe the is a hierarchy which we will say this officer.

R: No the hierarch is there, the disease it can fall directly under disease control and the officer report to me but all of them should report to the disease control officer any abnormality that they see.

N: Alright, so what responsibility if any do district health administration of LF and STH endemic areas like here have the control of these diseases.

R: All responsibility I’ve told you is basically that of coordination.

N: Alright, Okay, so what are the reporting channels for LF and STH epidemiological status and control activities in the district from affected communities to the regional level. The reporting channel?

R: When they are detected at the CHPS compound or in the community it is reported to the CHO in that area to in turn report to the sub district head and it will be reported to the district, but the issue is that before going to turn all formality they have to call directly so that we know if we have to take immediate action or not. The reason why it has to follow turn line is to ensure that whatever reporting is at the district level can be trace back into the community.

N: and then from the district also goes to region. Ok, so which officers are responsible for doing this reporting an LF and STH from the sub district level to the district level?

R: it’s the field technicians and community health officers.

N: so specifically when you take the district director like yourself in communities where LF and STH is endemic what will you say are their role in the achievement of the control objective for the district

R: The role of what the community?

N: Ok the district director in ensuring that the control objective for is achieved

R: its an important role if bring about a lot of complications and can even bring poverty so if you have reporting that they coming from a particular community, we need to do a spot map and then zoom in to take whether decision we have to take to ensure that we reduce the disease burden.

N: So, and when it comes to NTD program which is at the national level do they have any responsibility for ensuring that the control LF and STH is also achieved in the district, do have any particular responsibility when it comes to the control of disease in the district like here of example

R: when they come they say its from WHO or they say it is this one , so do I say its from them

**Section C: Financing**

N: Okay right, so how is NTD activities funded in the district with respect to general Ghana Health Service pooled funds or earmarked funds from the NTD program fund like we’ve mentioned

R: No the NTD has not special fund money that they bring, the money they bring are basically when they have from the donors

R: basically for mass drug administration

N: alright

R: So what we do is we try to integrate it a little bit by using the money for surveillance that comes from Ghana Health Service. So we go around looking for other disease , we put it also as part the things that we looking for in our surveillance system but there is no special money from anywhere .

N: okay, so I think that this question naturally follows, I will still ask it. Are there situations where NTD control activities are supported with general district health administration pooled funds from other priority disease intervention program such us, say reproductive health, TB, AIDS, Malaria?

R: We don’t say its NTD if its TB money, if its TB money we do TB activities, we add on some NTD activities but we don’t use the money to say that we are doing NTD activities rather than that other people will say misapplying the money

N: Misapplying the money in the same way if we turn the coin around, are there situation where the funds for NTD activities are also used to support other priority interventions?

R: No, for the NTD is very small I cannot do that.

**Section D: Planning**

N: Alright, we will quickly move to issues on planning. So what is the role of the district health administration in assessing resources needed for the control of NTDs in the district?

R: They would put it in our budget but the budget themselves we don’t get it. The budget is seriously under fund .we are talking from January to now there is no money from government activity and we rely basically on 10%,15% mark up on whatever service or drug given. The drug is very minimum and rely mainly on services which the IGF for general running of the health system.

N: So when it come for example like you mentioned if you have STH do you take part at the beginning of the year may be determining that this is how much we will require to appropriately to deal with conditions

**R: Oh we stopped doing it after a period because when you budget you don’t get the money you just see as part of the general health sector challenge**

N: What about the regional health administration, do they also do that assessment, do they assess the needs

R: I don’t know

N: so they don’t try to assess what each district requires?

R: I say don’t know

N: Okay and what is the role of NTD program in accessing the resources needed for NTD control activities in the district

R: Whether they assess it or not, I don’t know

N: what is the role of the district health administration in setting priority for NTD control activities in the district?

R: If we are to develop a skill it will be about *balist???* of the program

N: Alright, does the district health official set priorities and say this is what we are going to with respect to STH ?

R: Of course when you have a problem whether there is money or no money sometimes changing people’s behavior will just help so we do it

N: and what is the role of the regional health administration is setting priorities for the control of LMF and STH in the district?

R: The only invite us to workshop but they don’t talk much about mass drug administration and other things they don’t make too much noise about them

N: and when it comes to NTD program what role do they play in setting priority for the control of LF and STH in the district?

R: It is the matter of coordination

N: Okay especially coordinate what happens and determining the time when distribution is done for example.

R: That one I think is determine nationwide at the national level

N: what is the role of the district health administration in allocation of resources for NTD control activities in the district?

R: If you are not getting resources how do you allocate

N: but you mentioned that sometime you get something small and what do you play in allocation of this

R: No, the role that you play in allocation is paying of CBA community bases agent and fuel and all those things

N: do you determine what you pay them?

R: we don’t determine but there is a big challenge because for the past four to five years the money we have paid to Community Bases Agent is far lower than what we have paid polio volunteers so sometime people don’t want get involve and all the numbers, the numbers for volunteers for NTD are fewer than the number for polio

N: Mainly, because of how much they are paid differs

R: Apart from the differs the number are also small

N: what is role of the regional health administration in allocation of resources for LF and STH control activities in the district?

R: They bring the moneys to the region and they have their own formula that use in distribution, may be based on population, it may be based on how difficult it is to reach the population that is what I guess

N: For the NTD program do you think they also play role in the allocation of the resources for the national NTD program in the district

R: I don’t know

**Section E: Service delivery**

N: what category of staff are the district health administration works on the NTD control activities in the district

R: Basically technical officers

N: and then which organization or agency employed these staff who work on NTD control activities

R: Ghana Health Service

N: are these staff dedicated to NTD activities or they work on other Ghana Health Service activities?

R: They are program officers we just add NTD to their schedule

N: so they work on other ….

R: Yes you can’t employ someone for just NTD activities

N: do these staff also share office space, equipment, vehicles, stores with other staff of the district health administration

R: If the money is not enough how can they buy vehicles for the NTD programs. They use the general pool vehicles to carry out their activities

N: and the office space they share the same office?

R: The person is a technical officer, disease control officer has an office here and she runs the program concerning diseases and how to control them, she lives with her field technicians to do the job

N: Are any of the NTD control activities undertaken with other disease control in district

R: Yes, sometimes like I told with LF and malaria especially when we are doing talks on long lasting bed nets

N: What referral options are available for NTD cases identified in the peripheral health facilities in the district?

R: luckily we have a district office so they refer to the district hospital and if it is beyond them they refer to the regional hospital

N: what are the essential logistics and drugs required for NTD control activities in the district?

R: albendaziole, ivermectin,

N: those are drugs are there any logistics needed

R: we need fuel, we need those sticks for measuring height and we need money

N: so what is role of the district health administration in the procurement of drugs and logistics that used for NTD activities

R: we have no role in it

N: so what is role of the district health administration in the storage, transportation, distribution and delivery of the NTD drug and logistics to target population

R: we are actively involve because the drugs and we look at the community population and other thing to distribute and we distribute from here to the sub district and they distribute to the volunteers and we talk them about importance of storage and how it affect the quality of the drug

**Section F: Monitoring and evaluation**

N: Monitoring and evaluation. So what monitoring an evaluation activities are conducted by the district health administration to assess the achievement of NTD intervention objectives in the district, once we have been doing mass drugs administration in the district is there do any assessment ?

R: Yes we look at the report that is coming and check whether we are getting less cases of LF and also try to finalize like a I said earlier spot map to know which new areas or old areas are at a plan and then continue

N: are there laboratory base monitoring and evaluating activities that goes on to assist the district?

R: No

N: what ICT infrastructure, here I mean computers, internet access, website, telephone is available at the district health administration for processing, and storing and communicating NTD related information?

R: We use the DHIMS now

N: so you have internet access and then?

R: Yes all of them

N: alright and do the technical officers that you mentioned that they work on NTD activities , do they share this IT infrastructure with other staff?

R: its not all staff that enter into it and we meet to discuss it .all staff technical officers, disease control, nutrition and health information they are key in their part and we meet as a team to look and help us inform us on how to take decision

N: so what is role of the district health administration in data collection for NTD,M&E in the district

R: we ensure that data, we give the form for them to report on cases and we try to analyze the cases using OPD register CD1, CD2 forms

N: what is the role of the district administration in the analysis of these data that you get

R: there is no sense collecting data without analyzing it because you need to analyze the data to make decisions so analyze it

N: so they analyze at the district level. Alright when it comes to the regional health administration do they have any role M&E activities or LF and STH activities in the district?

R: Only during mass administration

N: what then do they do?

R: They come around to come and ask how many average drugs reaction cases have we seen that’s all

N: but when it comes to, you mention for example analyzing report to see do they?

R: I have never heard them analyzing any data

N: Okay but don’t they for example have to receive that information?

R: Yes, then it goes to the public

N: and the NTD control program what activities or what is their in role in M&E activities in the district , do they ever come to collect either blood samples something like that to find out ?

R: Is KCCR that do that is just recently Baaba has come and one girl from the school of public health but they are not directly to National Neglected Tropical Diseases they are not from that area

N: Okay they don’t come here to collect blood samples for example to examine to whether the MDA is effective?

R: Last year I think they came , Late last year and early this year I think is my first time I have seen them here in about eight years doing that but cases here is a cross – section study

N: so I think that I asked a question whether there was another organization that is involve in the control and you mentioned KCCR what exactly do they do

R: They are doing research, Its Kumasi Centre for some collaboration with Germans so they use doxycycline

N: They also try to control LF using doxycycline .

R: alright

**Section G: Demand Generation**

N: so the last section is on demand generation and social mobilization .what activities does the district health administration undertake to improve demand for the NTD intervention in the district

R: we do social mobilization, remind often school health to treat children and then also tell them about the importance of hand washing, other things.

N: which mediums for example do you use in this social mobilization activities, radio or which?

R: School health we go there, community debate

N: what is the nature of financial incentives available to staff of the district administration to improve coverage of the NTD intervention of the district?

R: No cash incentives

N: Are there any financial incentive from non-district health administration staff in the NTDs activities?

R: Yes if it is from mass drug administration you are paying some daily allowance

N: and which groups are you talking about particularly?

R: The volunteers who distribute the drugs.

N: alright, and what are the sources of this financial incentives for the volunteers

R: It comes from the ministry, maybe through WHO and other organizations that I don’t know.

N: so which staff of district administration are involve in developing materials and conducting health education and social mobilization activities in the district

R: Those already developed from the health promotion unit and that’s what we use .

N: Is it health promotion from national level?

R: we pick it from the regional level which they get from the national level.

N: which staff here are involve in the conducting of the social mobilization

R: The district director, the community health nurse and disease control officer

N: do this staffs also conduct social mobilization for other intervention other than NTD ‘s

R: Yes social mobilization and community talks equally health promotion is part of the work of the community organization

N: what is the role of health promotion unit in conducting social mobilization at the district level

R: we don’t have a health promotion unit

N: there is no health promotion unit at the district level, alright thank you very much.

**Section H: We are rounding up**

N: Please you don’t mind how long have you been a district director of Ahanta west?

R: I think four years

N: Before then what was your previous appointment

R: Acting district director and medical superintendent

N: was it in this same district?

R: No in this district and other district

N: So Dr. Sunderland thank you very much for your time and the invaluable information that you have provided for us

R: Thanks for coming

N:I’m very very grateful

I - Goodmorning once again, I’m Ernest Mensah, PhD student of the University of Ghana, School of Public Health. This study is part of my academic requirement and I want to thank you sincerely for making time out of your busy schedule to meet me and also to be interviewed for this study. I’m very grateful for your time and indeed your input as the Director. Your responses can be brief as possible but indeed elaborate where you think will be appropriate. The interview is expected to last at most an hour and it will be recorded and transcribed for data analysis. Please do you have any comments and questions?

R - No. I accept this interview and am very grateful to be part of this endeavor.

I-The questions are divided into sections, the NTDs situations, then we look at management issues, we look at service delivery, financing, planning, issues of demand generation and monitoring and evaluation. We’ll start with the (Neglected Top Diseases) NTDs situations in the district first. The first question is; Are NTDs amongst the priority diseases in the country?

R- Yes they are

I - Which NTDs are reported

R - we report on Onchocerciasis, we also report on schistosomiasis and we also report on filariasis. So these are some of the diseases that we report related to NTDs.

I - So for this study we we’ll be looking at those you’ve mentioned. Earlier on you mentioned that it is a priority disease. Is it priority because of the endemicity or the morbidity and its impact on the population?

R- It is the combination of all you’ve said that is how come we have included them as the top priority.

I - which of these NTDs are considered as endemic to the district?

R - Onchocerciasis and schistosomiasis are endemic but with the filariasis I’m no too sure

I – generally, what interventions are in place in the district to control these NTDs?

R- As far as onchocerciasis is concerned, we havedistribution of drugs to communities, thus, every year. It is a programme that goes on every year and there is a school health programme for schistosomiasis that goes on periodically and those who pass through the routine are identified and treated accordingly. So what it means is that the institution stocks the medicine so the diseases are treated as and when they come.

I – do you also undertake health education when it comes to this aspect?

R- Yes we do. The community health nurses are put into groups who routinely interact with the community by the nature of their works. They do that as part of the health education. They also make it part of their efforts during home visits. We planned to do it on air which will give as a further reach but has not being successful due to some constraints. So for now we are educating the communities through the community health nurses on these NTDs during their outreach programs.

I – So what are the general objectives or expected outcomes of what you have said?

R- first of all, is to create awareness and also remind people in the community about the impact of the diseases and how disabling the diseases and therefore to embrace the interventions of what we have in place in terms of community drug distributions and for those who have overt manifestation of schistosomiasis we expect that they come to the hospital and also to avoid contact with the sources of the causative agents. But as far as onchocerciasis is concerned, as I indicated earlier, it is endemic. We ensure that, when you catch the causative agent, we can deal with it. There no need to separate the agents and the host.

I – for the intervention on the drug distribution per-se, do you have any objectives for undertaking them?

R- the objective is to reach a proportion of the target Group. We try to hit about 80% of the group and I think over the years we’ve been able to realize this goal. Occasionally, we have people reacting to the conditions and there is a system in place before the drug distribution where they are encouraged to visit the nearest health facility when there is a problem and they are treated free of charge. They are given preferential treatment where they are not allowed to go through the waiting period. So these are some of the objectives. However the main objective is to hit 80% of the population.

I – when it comes to the control of these diseases, do you have any objectives?

R- No, we do not have any objectives with respect of the incidence of the diseases.

I - So does it mean the education and the distribution of drugs were initiated by the NTD programme?

R- Long before the NTD programme this whole thing was in existence so we can say the NTD programme is lending a support for an existing programme. Because the nature of the programme needs external help and I think that is what the NTD programme is doing.

I - do you have any timeline with respect to when to stop or not in view of the objectives.

R- No, we do not have. In fact we do not intend to stop. There is a spectrum of interventions and we have taken a band of that spectrum. So when we stop we might end up not achieving anything. So the drug distribution must go along other intervention.

I – So are there other intervention beyond the ones you’ve already mentioned?

R- I can’t really tell whether there has being other interventions.

**Stewardship and Governance**

I - Let’s look at issues of stewardship and governance. What is the role of the district health administration in the control of the NTDs?

R- Ours is to coordinate the activities, it is also to organize and also to train the human resource to make sure that the distribution of drugs go according to schedules. And also to avoid unnecessary shortages during distribution. And also the target group embraces the idea. We also invite other agencies to assist. Agencies that are good in advocacies as well as those who can help financially.

I – what is the role of the NTD programme in the districts?

R- Mainly, it is to bring in additional funds/resources. That is essentially what they do but the resources are used for different activities.

I – which agency/organization do you consider ultimately responsible for the control of these NTDs?

R- the Ghana Health Service

I – looking at the NTDs in the district, do you consider the district as ultimately responsible or the NTD programme or the Region

R- the district is ultimately responsible for the control in the district but we are responsible and accountable to. But we also in need of resources to as to enable us put on the ground what we put on paper.

I – which officer(s) would you say is ultimately responsible for the control of NTDs in the district?

R – the district director is ultimately responsible. But as you know he works with a team. The disease control officers, the field technicians as well as other colleagues who are doctors.

I – what are the reporting channels of NTDs from the communities up to the regional levels?

R- there are volunteers in the communities with supervisors. It may happened that a number of communities may have one supervisor of which they report to at the sub-district and he will compile it to the disease control officer at the district level and be sent to the regional level.

I – what criteria are these supervisors at each level?

R- the designation vary. There are community health services, there are physician assistants at the sub-district level and then we have the disease control officer at the district level but between the interfaces of these two, there are coordinators. These coordinators could be hospital staffs, nutrition officers or public health nurses who serve as a link between them.

I – what is the role of district officers in the areas where the NTDs are endemic?

R- They ensure there is requisite amount of drugs. He ensures that the sensitization of public health service is done properly. He ensures that the policies and plans are implemented fully to achieve the needed result.

I – what exactly do the district directors do to achieve the objectives of the NTD programme.

R- they play a supportive role. They imparts into the officers the manner in which the work they are doing is required. They inspire, motivate and teach the officers.

I – what generally are the sources of fund for the NTD programmes?

R- it comes from the programme itself, solicit for funds from the district assembly as well as the hospital.

I – what about Ghana health service funds?

R- Ghana health service funds are year mark funds. They are used for intended purposes

I – Has there be a situation where these activities could be used to develop other priority interventions in the district?

R- Yes we do that. We couple our activities with other resources because we know that we are resourcedtrapped. We therefore look for other related activities that we might be able to curtail alongside the NTDs.

I – does it apply the other way round where other funds are used to fund the NTDs programme?

R- yes. We sometimes even prey finance. As a result of the delays in finance. We use other funds to do it knowing that we will pay later.

I – Has there being a situation where other organizations fund the project?

R- No, it’s only the assembly.

I – what is the role of the district health administration in assessing the resources that are needed in the implementation of the NTDs.

R- In planning we are able to gauge the resources that we will require for the whole activities, the district health activities. It helps the district to identify when the resources fall short.

I – what about the regional?

R- The region also helps in terms of transport because our area has a difficult terrain, some of the villages you have to pass through Tarkwa, Bogosu and prestea. The region also sometimes helps us.

I - Does it mean they make an estimate of what must be done

R- Yes they do

I- what about the NTD programme?

R- They bring their funds and the funds do not consider the number ofvolunteers,

I - What role has the district health administration play in setting priorities with respect to this NTD programme.

R -In terms of priority, we do exactly what the NTDs programme suggest.

I - So it means that the priorities are set by the NTDs.

R- Yes.

I –what about the regions too?

R- I may not know if they do because they did not

I – what is the role of the district health administration in the allocation of NTDs programme activities in the district?

R – The district health administration sees to it that every district receives fair distribution of the resources which has been set out.

I – do the regional health administration play any role in the allocation of resources for NTDs programme activities.

R – They give a spreadsheet which guide in the allocation of resources. Often the allocation is based on certain criteria, on of this is Population distribution. But when we are considering the terrain, the accessibility we know that though the population of a particular group is not big, they might need more of the resources because of the difficulty in the area.

I – does the NTD programme play any role in the resource allocation?

R – Yes, the NTD programme allocates the resources based on fact and figures we’ve given them; the target group, the number of sub districtand also the duration of the project.

I – What category of staff at the district health administration work on the NTDs activities?

R – Everybody. The health information officer does that, the field technician as well as the disease control officer and the accountants. All these people are delegated by the Ghana Health service.

I – so it means that these people are not dedicated to the NTD programmes.

R – Yes, they perform multi-purpose activities.

I – are any of the NTD activities under taken alongside any other disease activity in the district?

R – Yes, I think we’ve talked about TB.

I – Are there other referral system for the control of NTDs in the district?

R – Yes, there is a referral system for diseases. Normally, depending on how the disease comes to our notice. If the disease comes to our notice through the community health service, and it’s beyond their control, they refer to the hospitals for treatment.

I – what are the essential logistics and drugs available for the treatment of NTDs?

R – Vehicles, we also need the appropriate drugs. Apart from this we also need to train people and retrain peopleas well as sensitizing the people through adverts on radios, and other public address systems.

I – does the district Health administration play any role in the procurement of these drugs?

R – No, we essentially receive; we don’t also determine the quantity of drugs to be procured.

I – Does the District assembly play any role in the storage, transportation and the distribution of these drugs?

R – Yes we do. We as the district receive the drugs from the region where we give it to the sub-district and it is given to the communities through the volunteers in the community. Normally, the district reserves some amount of the drugs to take care of any shortage that may rise. We store the drugs in the district store.

I – What monitoring and evaluation activities are conducted by the district health administration (DHA) in assessing the achievement of the intervention objectives of the NTDs?

R – The supervisors are supposed to monitor compliance to dosage; whether the appropriate dosage is used. Onchocerciasis, for instance we use height. The supervisors normally use samples to make sure that the right thing is done. They consider the recording of which they then transcribe it and send it to the disease control board. There is always a team that preforms a survey to see the coverage whether it matches with the plan of the district.

I –Does the NTD programme itself preform any survey to know the impact of the programme?

R – There are officers who normally come around to check

I – when the officer comes to perform the survey, does the district contribute?

R – Before they come, they inform us since they do not know exactly what they are coming to do. They also have to make sure those they are coming to meet are there.

I – what ICT programmes are put in place to help in the dissemination of information about the NTD activities?

R – People have access to the internet and other ICT programmes which help in the dissemination of information in a timely manner.

I – Are these ICT structures for the NTD programmes used for any other purposes?

R – Yes, they are. Some of the ICTs, we had them from the region whiles the district procure the others.

I – what is the role of the DHA in the collection of data to assess the intervention of the NTDs activities?

R – The district collects data. For instance when we distribute drugs, we perform data collection of the coverage in the communities of which we use to analyze our performance.

I – what role does the regional Health administration play in the monitoring and evaluation of the NTDs intervention?

R – The region has a supervisory role. They also consider their data quality.

I – how is this data quality carried out?

R – They look at the figures that might not be matching and they give us the feedback.

I – what duty does the DHA performs to improve coverage of the NTD programmes?

R – The district sensitizes the communities beforehand in order to accept the officials when they come around. We also educate the community health nurses to interact with the community members. We sometimes use the radio for adverts. The community also sometimes beat the gon-gon or has their own PA systems that they use.

I – what is the nature of the finance available for the district to improve NTDs intervention?

R – We ensure that the district assembly and the hospital to increase their financial support. That is the only way we can increase the coverage. There are reward systems out in place for an officer who performs that activity and that reward is regular.

I –what is the source of these resources?

R –from the NTD programme, the Hospitals

I – what is the nature of the incentives given to non-administration members of the district?

R – They get the same amount with respect to the officials of the district provided the nature of work is the same

**Demand Generation**

N: We are on the last section,the demand generation,so what activities does the district health administration undertake to improve coverage and increase demand for NTD interventions activities in the district?

R: The district does a lot in terms creating awareness, sensitizing people. When we are going to have a program like that we keep the community informed beforehand, maybe two weeks before hand so that they would be ready to receive volunteers when they come. So that is essentially what the district does and as I alluded to earlier, we use our community health nurses who come into direct contact with them. We use the existing communication system in the community so they beat gongon they use their own PA system and we also use the fm to get them mobilize.

N: So what is the nature of financial incentives available to staff of the district health assembly to improve coverage of NTD interventions?

R: The part we can do is to ensure that the district assembly and the hospitals are giving us more resources, because as for the NTD theirs is a given so perhaps we can’t do much but for the district assemblies and the district hospitals they can raise their level of support the funds that they use to support us with. So once they do that we can also do more to increase the coverage.

N: So are there some financial incentives for the staffs to encourage them to work so that coverage is increased

R: The existing reward system is the regular reward system, there is an allowance that is given to an officer depending on the nature of his work and that is regular. So that is what is done that is what is done, we don’t do more than what I said.

N: And what is the source of these resources that is used to motive the staffs?

R: As I said earlier, they come from the NTD, they come from the district assembly, and they also come from the hospitals as IGF to support us.

N: and then when it comes to, am aware that some non district health administrative staff take part in this activities, what form of financial incentives do they also get?

R: They get the same as the regular health staff gets so long as those whose nature of work are the samething they get the same level of allowance regardless of where you are coming from whether you are a Ghana health service staff or non Ghana health service staff so long as you are a supervisor you get what the supervisor get, so long as you are a coordinator you get what the coordinator gets so long as you are a driver you get what a driver get, and it has a margin this is a kind of intervention that require mobilizing resources beyond what the district health administration can master so we tap into other decentralize agents to give us vehicles to put their drivers at our side, so we ought to treat them well so that they can embrace subsequent programs.

N: Could you mention some of the non NTD staffs that you use?

R: Agric staffs we use the Ghana health education staffs we use non formal community staffs these are the, we even use the district the national health insurance staffs to do the work.

N: and for this incentives that you give them also, from what sources do they come from?

R: The same source.

N: So when it comes to this sensitization activities and health education which staff at the district health administration does that, yes conduct that?

R: The health information officer does that, we have very experience field technicians they also do that and the disease control officers. Sometimes we enlist support from the assembly, there is this group and decentralized agencies that work with the communities regularly so they know the community nuances. When we explain to them exactly what we want/objective of what we want. They go with our officers and give the message.

N: Do these staff you have mention like disease control officer, information officer, field technicians develop materials, conducting health educations and social mobilization activities in the district

R: Yes they do. Actually the district has a social mobilization and community sensitization materials, it is standardized. So they all have the exact message to give out to the communities. The posters, flyers are develop above us we receive from region and I would have taught the region also receive from national level.

N: Is there a health promotion unit in the district health administration and what role do they play in conducting social mobilization activities.

R: There is no specific health promotion unit but there are various category of health officers who are involve in health promotion .You know because of the nature of health system at the district level, various category of people play roles beyond what is normally required of them .So a nutrition officer would not be concentrated on only nutrition he would be involved in prevention, health promotion and the rest of them. So they are a number of health officers at the district , who are involved in health promotion activities.

**Section H: We are rounding up**

N: How long have you been a district director in this district?

R: Seven years.

N: Before then what was your previous appointment?

R: The same district director I was at Enchi

N: Thank you very much for this interview and I am very grateful for your time. Do you have a comment or questions before?

R: No, I am very happy. Very happy in the sense that you have explored various aspects of the work and as challenged me to look into those areas very well and also share with my colleagues. The officers I work with about the areas that we have dealt with and how we can improve the nature of work relative to those areas. So it has been a very useful encounter and I am also happy

N: I also thank you.
